# Hydrogels for Local Drug Delivery in Biofilm-Associated Periprosthetic Joint Infection: Current Progress and Future Directions

**DOI:** 10.3390/microorganisms14091882

**Published:** 2026-08-24

**Authors:** Karolina Kraus, Paweł Mikziński, Bindu Subhadra, Emil Paluch

**Affiliations:** 1 Faculty of Medicine, Wroclaw Medical University, Wybrzeże Ludwika Pasteura 1, 50-376 Wroclaw, Poland; karolina.kraus@student.umw.edu.pl (K.K.); pawel.mikzinski@student.umw.edu.pl (P.M.); 2 Department of Biological and Earth Sciences, Arkansas Tech University, Russellville, AR 72801-8805, USA; 3 Department of Microbiology, Faculty of Medicine, Wroclaw Medical University, Tytusa Chalubinskiego 4, 50-376 Wroclaw, Poland

**Keywords:** hydrogels, antibiotic-loaded hydrogels, anti-biofilm enzymes, phage therapy, smart hydrogels, nanoparticle-loaded hydrogels, periprosthetic joint infection, biofilm infections

## Abstract

Periprosthetic joint infection (PJI) remains one of the most serious complications of arthroplasty, largely due to the formation of microbial biofilms on implant surfaces. Biofilm-associated infections exhibit increased tolerance to antimicrobial therapy and host immune responses, making eradication difficult and often requiring repeated surgical interventions. Consequently, there is a growing need for effective local therapeutic strategies capable of delivering high concentrations of antimicrobial agents directly to the site of infection while minimizing systemic toxicity. Hydrogels have emerged as promising drug delivery platforms for the management of biofilm-associated PJI. Their biocompatibility, injectability, high water content, and tunable physicochemical properties enable controlled and localized release of therapeutic agents within the infected peri-implant environment. This narrative review summarizes recent advances in hydrogel-based approaches, including antibiotic-loaded hydrogels, systems incorporating anti-biofilm enzymes, bacteriophage-loaded formulations, and nanoparticle-enhanced platforms. It also highlights future research directions, with particular emphasis on the need for expanded clinical studies to facilitate the translation of emerging hydrogel-based therapies into clinical practice. Further development of these systems should focus on the incorporation of novel therapeutic agents into hydrogel platforms, aiming to enhance biofilm eradication and improve treatment outcomes in patients with PJI. Particular attention is given to stimuli-responsive (“smart”) hydrogels that release therapeutic payloads in response to infection-related triggers such as pH changes, with emphasis on the need for expanded clinical studies to facilitate the translation of emerging hydrogel-based therapies into clinical practice. Further development of these systems should focus on the incorporation of novel therapeutic agents into hydrogel platforms, aiming to enhance biofilm eradication and improve treatment outcomes in patients with PJI.

## 1. Introduction

Periprosthetic joint infections (PJIs) remain one of the major complications associated with orthopedic implant surgery. Despite advances in biomaterials and antimicrobial implant technologies, prosthetic surfaces remain susceptible to bacterial adhesion and biofilm formation, particularly by staphylococcal species. The increasing prevalence of antimicrobial-resistant pathogens further complicates treatment and limits the effectiveness of conventional antibiotic therapy [1]. PJIs are classified as acute or chronic infections, with acute PJIs presenting within four weeks of symptom onset, including early postoperative and acute hematogenous infections, whereas chronic PJIs are characterized by symptoms persisting beyond four weeks.

In early PJIs, debridement, antibiotics, and implant retention (DAIR) remain the preferred treatment strategy when implant retention is feasible; however, its success depends on multiple factors, particularly the timing of intervention [2,3]. For chronic infections or cases unsuitable for implant retention, two-stage exchange remains the standard treatment approach, although alternative strategies such as one-stage and 1.5-stage revisions are increasingly investigated to improve outcomes and reduce treatment burden [4]. Despite advances in surgical management, PJIs remain associated with high rates of treatment failure, prosthesis loss, and significant patient burden.

Therefore, novel approaches aimed at improving local antimicrobial delivery are being explored. Hydrogels represent a promising platform capable of providing localized, sustained release of antimicrobial agents directly at the infection site, with potential applications in both PJI prevention and treatment. This review summarizes the current applications, advantages, and limitations of hydrogel-based strategies for managing PJIs.

## 2. Hydrogels Used in PJIs

Hydrogels are three-dimensional, hydrophilic polymer networks capable of retaining large amounts of water or biological fluids within their structure without dissolving. This unique property arises from the presence of crosslinked polymer chains that maintain structural integrity while allowing significant swelling in aqueous environments. As a result, hydrogels exhibit soft, tissue-like mechanical characteristics, which makes them particularly suitable for biomedical applications. In a biological context, hydrogels can mimic the physical properties of the extracellular matrix, providing a supportive environment for cells and enabling controlled transport of water, nutrients, and bioactive molecules [5].

In the context of PJI, hydrogels have been investigated for several complementary applications, including antimicrobial implant coatings, local treatment of the peri-implant space and bone defects, and as temporary matrices supporting localized antimicrobial therapy [6,7]. When applied as a coating, hydrogels can form a temporary hydrated barrier on the implant surface, which may reduce bacterial adhesion and subsequent biofilm formation. Clinically investigated hydrogel coatings, such as the fast-resorbable Defensive Antibacterial Coating (DAC^®^), can also be applied to prosthetic components during primary or revision arthroplasty and may be used alone or loaded with antibiotics to provide additional protection against bacterial colonization [8,9]. In the treatment of established PJI, antibiotic-loaded hydrogel coatings have been evaluated as an adjunct to surgical management, including one-stage and two-stage revision procedures, with the hydrogel applied directly to the implant surface to provide localized antimicrobial activity [10]. Hydrogels may also be used to fill irregular peri-implant spaces or surgically debrided areas, thereby providing a local matrix for the delivery of antimicrobial or anti-biofilm agents. More advanced experimental formulations have incorporated bacteriophages, nanoparticles, enzymes, and other anti-biofilm components, expanding the potential role of hydrogels beyond conventional antibiotic delivery [6]. However, despite promising clinical and experimental results, the evidence supporting hydrogel-based approaches in established PJI remains limited, and these systems are currently considered adjunctive strategies rather than replacements for surgical debridement, implant management, and systemic antimicrobial therapy.

**Figure 1:**
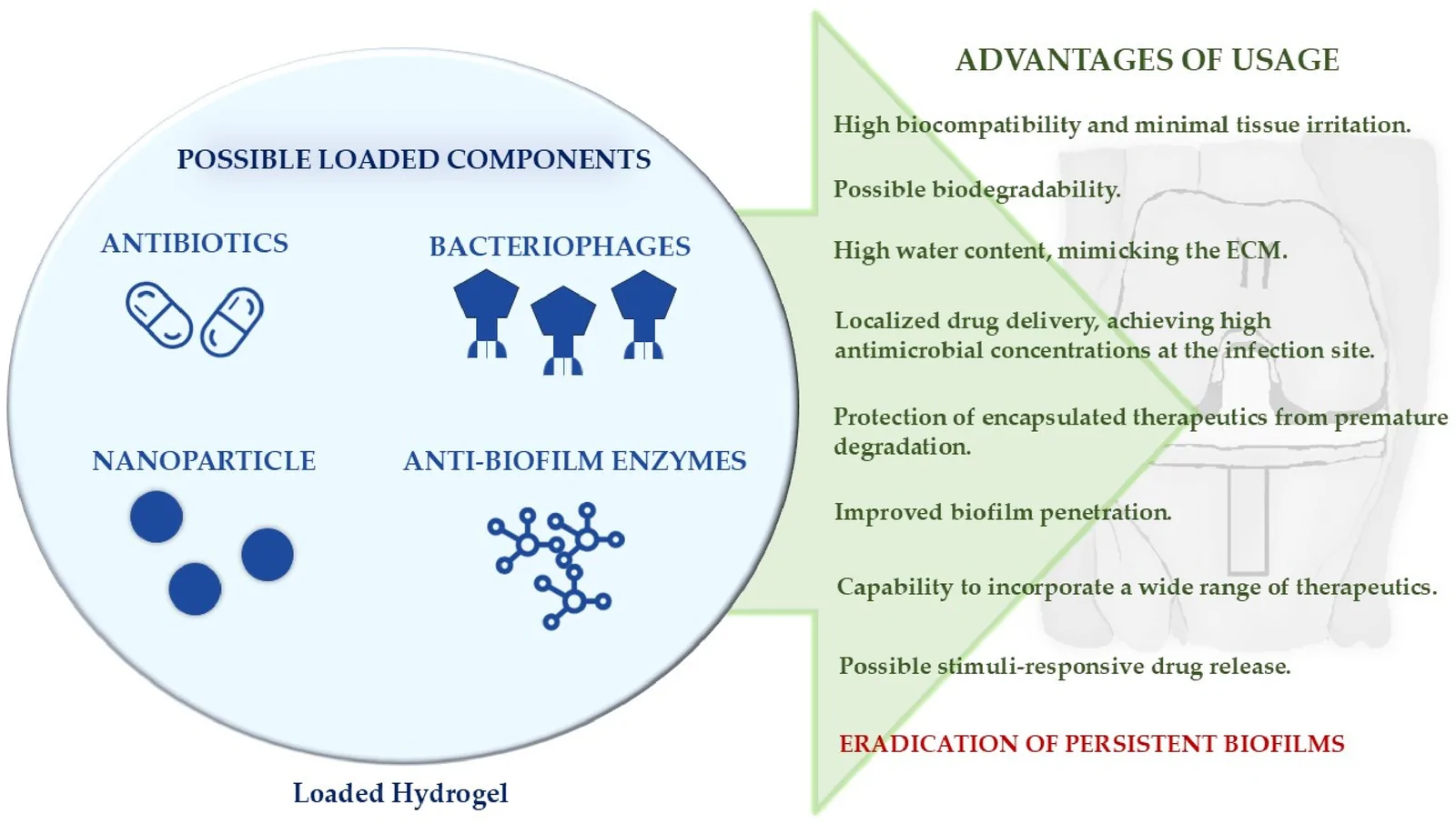
Schematic illustration of a hydrogel-based platform for biofilm eradication. Hydrogels can be loaded with various antimicrobial agents, including antibiotics, bacteriophages, nanoparticles, and anti-biofilm enzymes. Their high biocompatibility, biodegradability, ability to provide localized and stimuli-responsive drug delivery, protection of encapsulated therapeutics, enhanced biofilm penetration, and capacity to incorporate diverse therapeutic agents make them promising systems for the eradication of persistent biofilms.

Hydrogels are especially promising biomaterials for local drug delivery in PJI due to their high water content, excellent biocompatibility, and highly tunable physicochemical properties. They can be engineered as fully resorbable carriers with controllable degradation rates and drug release kinetics, enabling sustained delivery of antimicrobial agents directly at the infection site [6]. Their soft, injectable consistency allows effective application to implant surfaces and filling of peri-implant space, while also supporting adaptation to the infected microenvironment. Hydrogels can usually be prepared under mild conditions, avoiding thermal degradation of incorporated therapeutics and enabling the incorporation of a broad spectrum of agents, including antibiotics, antifungals, bacteriophages, and anti-inflammatory compounds [11]. Experimental studies have further demonstrated their ability to achieve bactericidal concentrations against common orthopedic infection pathogens, while advanced formulations allow for multi-phase and prolonged release of multiple active agents, including simultaneous analgesic delivery [6]. Overall, hydrogel design for PJI is highly versatile and can be systematically tailored by modifying key parameters such as polymer origin, crosslinking mechanism, degradability, stimuli-responsiveness, and physical form, all of which critically determine their performance as local drug delivery systems [12]. The main advantages of using hydrogels against biofilms are presented in Figure 1.

This section provides a structured overview of the main classification criteria of hydrogels relevant to PJI applications, with emphasis on their functional implications for clinical use. All information presented in this chapter is summarized in Table 1.

### 2.1. Classification Based on Polymer Origin

Hydrogels intended for biomedical applications are commonly classified according to the origin of their polymeric network into natural, synthetic and hybrid systems.

Natural hydrogels are composed of biopolymers that closely mimic the composition and architecture of the extracellular matrix (ECM), thereby providing a biologically favorable microenvironment for cellular adhesion, proliferation, and tissue regeneration. Frequently employed natural polymers include alginate, chitosan, collagen, gelatin, hyaluronic acid, and fibrin. Owing to their inherent biocompatibility, low cytotoxicity, and susceptibility to enzymatic degradation, these materials have been extensively investigated as carriers for localized drug delivery, particularly in orthopedic and regenerative medicine applications [13]. Nevertheless, their clinical translation remains constrained by several intrinsic limitations, including relatively poor mechanical strength, uncontrolled degradation kinetics, limited structural stability, and batch-to-batch variability associated with biological sourcing [14].

In contrast, synthetic hydrogels are fabricated from chemically defined polymers, such as poly(ethylene glycol) (PEG), poly(vinyl alcohol) (PVA), polyacrylamide (PAAm), and various biodegradable synthetic macromolecules. The principal advantage of synthetic polymer networks lies in the precise control that can be achieved over their physicochemical properties, including crosslinking density, porosity, swelling capacity, degradation rate, mechanical integrity, and drug release kinetics. Such tunability enables the rational design of hydrogel systems tailored to specific therapeutic requirements and clinical indications. However, synthetic polymers generally lack intrinsic biological functionality, including cell-recognition motifs and bioactive signaling domains, thereby often necessitating chemical functionalization or incorporation of biologically active components to improve cellular interactions and tissue integration [15,16].

To overcome the inherent limitations associated with either polymer class, hybrid hydrogels have been developed by integrating natural and synthetic polymers within a single polymeric network. Hybrid hydrogels are polymeric networks composed of at least two structurally or functionally distinct components, which may differ in chemical composition, morphology, or scale. They can be formed through the integration of natural and synthetic polymers, nanogels, or functional nanoparticles, including magnetic or carbon-based structures, connected via covalent or non-covalent interactions. Hybridization may occur at molecular or microscale levels, depending on the system design. Their modular structure enables the combination of mechanical strength, biological activity, and functional responsiveness within a single platform, overcoming limitations of individual materials [17]. As a result, hybrid hydrogels have gained increasing attention for biomedical applications, particularly in controlled drug delivery and regenerative medicine. Advanced fabrication approaches such as click chemistry, 3D printing, and photopatterning further enhance their versatility and applicability [18,19].

Collectively, the origin and composition of the polymer network constitute fundamental design parameters governing the physicochemical behavior, biological performance, and therapeutic efficacy of hydrogel-based drug delivery systems. Hybrid hydrogel platforms are increasingly recognized as next-generation biomaterials with considerable potential for the localized treatment of biofilm-associated periprosthetic joint infection.

### 2.2. Classification Based on Crosslinking Mechanism

Hydrogels can also be classified according to the mechanism of polymer network formation into physically and chemically crosslinked systems. The crosslinking strategy is a key determinant of hydrogel performance, governing mechanical strength, stability, degradation behavior, swelling properties, and drug release kinetics.

Physically crosslinked hydrogels are formed via non-covalent interactions, including hydrogen bonding, ionic and hydrophobic interactions, crystallite formation, and chain entanglements. Their reversible nature enables mild fabrication conditions, making them suitable for the encapsulation of cells and bioactive molecules. In addition, these systems may exhibit injectability and self-healing properties. However, they are generally characterized by limited mechanical stability, faster degradation, and less predictable release profiles [20].

Chemically crosslinked hydrogels are based on covalent bond formation through mechanisms such as photo-crosslinking, Schiff base chemistry, click reactions, and enzymatic crosslinking. These networks provide superior mechanical integrity, enhanced structural stability, and more controlled degradation kinetics, enabling sustained and localized drug delivery [21].

Overall, crosslinking chemistry represents a critical design parameter enabling precise tuning of hydrogel properties for targeted biomedical and drug delivery applications [22].

### 2.3. Classification Based on Degradability

Hydrogels used in biomedical applications, including the management of PJI, can be classified according to their degradability into biodegradable and non-biodegradable systems. This parameter is particularly relevant in PJI therapy, where sustained local antimicrobial delivery must be balanced with implant-associated safety and the avoidance of additional surgical interventions.

Biodegradable hydrogels undergo controlled degradation under physiological conditions via hydrolytic or enzymatic mechanisms, yielding non-toxic by-products that are eliminated through natural metabolic pathways. In PJI management, such systems are advantageous as they provide temporally controlled local drug release directly at the infection site and subsequently undergo complete resorption, thereby eliminating the need for surgical removal. Furthermore, their degradation kinetics can be tailored to match the duration of antimicrobial therapy, which is critical in biofilm-associated infections.

In contrast, non-biodegradable hydrogels retain their structural integrity over prolonged periods and exhibit minimal in vivo degradation. They function as long-term drug depots, enabling sustained antimicrobial release within the peri-implant space. However, their persistence may pose clinical limitations, including the potential for bacterial recolonization and the possible need for secondary surgical removal [23,24,25].

### 2.4. Classification Based on Responsiveness

Hydrogels can also be classified according to their responsiveness to external or internal stimuli. This category is particularly relevant in the context of PJI, where the infected microenvironment is characterized by dynamic biochemical changes associated with inflammation and biofilm activity. Stimuli-responsive hydrogels, often referred to as “smart” hydrogels, are designed to undergo predictable physicochemical changes in response to specific triggers, enabling controlled and on-demand drug release at the site of infection. Conventional (non-responsive) hydrogels release their therapeutic cargo primarily through passive diffusion and matrix degradation. In contrast, smart hydrogels respond to specific environmental cues such as pH variations, enzymatic activity, reactive oxygen species (ROS), or temperature changes [26]. These triggers are particularly relevant in PJI, where bacterial metabolism and host immune responses create a locally altered microenvironment compared to healthy tissue. pH-responsive hydrogels exploit the acidic conditions typically observed in infected and inflamed tissues, leading to structural changes in the polymer network and accelerated drug release. Enzyme-responsive systems are designed to degrade in the presence of bacterial or host-derived enzymes, while ROS-responsive hydrogels react to elevated oxidative stress levels commonly associated with chronic infection. Thermo-responsive hydrogels, on the other hand, undergo sol–gel transitions in response to temperature changes, allowing minimally invasive injection followed by in situ gelation at body temperature [27].

The ability of these systems to respond to infection-specific stimuli offers significant advantages in potential PJI management, including spatially and temporally controlled antibiotic delivery, improved targeting of biofilm-associated bacteria, and reduced systemic exposure. As a result, stimuli-responsive hydrogels are increasingly recognized as promising platforms for enhancing local therapeutic efficacy in orthopedic infections [28].

### 2.5. Classification Based on Physical Form

Hydrogels can also be classified according to their physical form and mode of administration, a parameter of particular relevance in the clinical management of PJIs. The physical format critically influences handling characteristics, anatomical adaptability, and suitability for minimally invasive orthopedic procedures.

Injectable hydrogels are among the most clinically relevant systems for PJI therapy. They are typically administered as low-viscosity precursors that undergo in situ gelation, enabling conformal filling of irregular peri-implant defects and infected bone cavities. This minimally invasive delivery is particularly advantageous in procedures such as debridement, antibiotics, and implant retention (DAIR), where localized therapy is required without extensive surgical exposure [29].

Preformed hydrogels are fabricated ex vivo as solid or semi-solid constructs and subsequently implanted into the defect site. They provide defined geometry and improved structural integrity but generally require more invasive surgical placement and offer limited adaptability to complex anatomical spaces.

In situ gelling hydrogels constitute an intermediate class, being delivered in a liquid or semi-liquid state and undergoing gelation in response to physiological triggers such as temperature, pH, or ionic strength. This approach enables precise localization at the infection site while ensuring adequate defect filling and intimate contact with infected tissues and implant surfaces [30,31]. However, their application in PJIs remains not extensively studied.

Overall, hydrogel physical form is a key determinant of clinical applicability in PJI. Injectable and in situ gelling systems are particularly advantageous due to their minimally invasive administration and conformability, whereas preformed constructs are more suitable when mechanical stability and defined geometry are required.

### 2.6. Challenges in the Clinical Translation of Hydrogel-Based Antibacterial and Anti-Biofilm Systems in PJI

Despite promising preclinical results and the availability of early clinical evidence for selected hydrogel-based antibacterial systems, several challenges limit their broader clinical translation. First, the substantial heterogeneity of hydrogel compositions, polymer architectures, crosslinking mechanisms, degradation profiles, and incorporated antimicrobial agents makes standardization difficult and limits direct comparison between studies [32]. Second, the performance of hydrogel systems observed under simplified in vitro conditions may not accurately reflect the complex clinical environment of PJI, where protein adsorption, inflammatory processes, synovial fluid, tissue interactions, mechanical loading, and interactions with implant surfaces may alter hydrogel swelling, degradation, antimicrobial activity, and drug-release kinetics [33]. Another important challenge is achieving a predictable balance between therapeutic persistence and biodegradation. The hydrogel should remain at the target site for a sufficient period to provide effective antimicrobial or anti-biofilm activity while undergoing controlled degradation without excessive swelling, premature loss of structural integrity, accumulation of degradation products, or adverse tissue reactions. For implant-associated applications, adequate adhesion and mechanical stability are also required to maintain the hydrogel at the intended site without compromising its function during implantation and subsequent mechanical loading. The complexity of multifunctional hydrogel systems represents an additional translational barrier. Although incorporation of antibiotics, nanoparticles, antimicrobial peptides, enzymes, bacteriophages, or other anti-biofilm agents may enhance therapeutic efficacy, increasing formulation complexity can introduce additional challenges related to stability, sterilization, manufacturing reproducibility, and quality control. Regulatory considerations may be particularly demanding for multifunctional systems combining a biomaterial with antimicrobial or biological agents, as these products require rigorous characterization, safety assessment, reproducible manufacturing, and appropriate sterilization procedures [34,35]. Finally, although clinical studies of antibiotic-loaded hydrogel coatings such as DAC^®^ have demonstrated feasibility and promising outcomes, the available clinical evidence remains limited, and systematic reviews have highlighted the need for larger and better-designed prospective studies to establish their long-term efficacy and safety in PJI [7,36]. Therefore, future clinical translation will require standardized hydrogel formulations and testing protocols, clinically relevant biofilm models, rigorous evaluation of long-term safety and degradation, reproducible manufacturing processes, and well-designed multicenter clinical trials.

## 3. Hydrogels for Local Antibiotics Delivery in Treatment of PJI

As discussed previously, the treatment of PJIs remains highly challenging, primarily due to the ability of microorganisms to establish mature biofilms on implant surfaces. Although prolonged systemic antibiotic therapy constitutes a cornerstone of current treatment protocols, its efficacy is often compromised by the limited penetration of antimicrobial agents into the biofilm matrix, poor vascularization of infected peri-implant tissues, and the inability to maintain sufficiently high drug concentrations at the implant–tissue interface. Consequently, systemic administration alone frequently fails to eradicate biofilm-associated bacteria and may require prolonged treatment, increasing the risk of systemic adverse effects and antibiotic-related toxicity [1].

To overcome these limitations, considerable attention has been directed toward local antibiotic delivery systems capable of achieving high antimicrobial concentrations directly at the site of infection while minimizing systemic exposure. Among these, hydrogel-based carriers have emerged as particularly promising platforms owing to their injectability, biocompatibility, biodegradability, and capacity for controlled and sustained drug release. By maintaining therapeutic antibiotic concentrations within the infected tissue for extended periods, hydrogels may improve bacterial eradication, reduce the likelihood of recurrent infection, and enhance the overall effectiveness of surgical management strategies such as debridement and implant retention or staged revision procedures [5,6]. Consequently, numerous hydrogel formulations incorporating conventional antibiotics have been developed and evaluated in both in vitro and in vivo models of PJI.

### 3.1. Mechanisms of Controlled Antibacterial Release from Hydrogel Platforms

Drug release from hydrogels is primarily governed by three main mechanisms: diffusion-controlled, swelling-controlled, and chemically controlled release. In diffusion-controlled systems, the therapeutic agent diffuses through the aqueous channels and pores of the polymeric network, with the release rate being influenced by factors such as polymer mesh size, drug molecular weight, and drug–polymer interactions. In swelling-controlled systems, water uptake causes expansion of the polymer network and increases the free volume available for drug diffusion, thereby facilitating drug release. In chemically controlled systems, drug release is associated with chemical changes within the hydrogel matrix, including polymer degradation, hydrolysis, or cleavage of labile bonds, which progressively liberate the encapsulated therapeutic agent [37].

These mechanisms may occur simultaneously within a single hydrogel system. In addition, external or biological stimuli, such as temperature, pH, reactive oxygen species, or enzymatic activity, can modulate hydrogel swelling, degradation, or drug–polymer interactions and thereby regulate drug release—mechanism of novel smart hydrogels. Depending on the physicochemical characteristics of the hydrogel and the loaded agent, these mechanisms can result in different release profiles, including an initial burst release followed by sustained or prolonged drug release [38].

### 3.2. Single Antibiotic-Releasing Hydrogels

In a study by Liao et al. [39], a vancomycin-loaded hydrogel was developed based on oxidized hyaluronic acid (oHA) crosslinked with adipic acid dihydrazide (ADH). The system was comprehensively evaluated in terms of degradation behavior, drug release kinetics, cytocompatibility, and antimicrobial activity. The oHA–ADH hydrogel exhibited an initial burst release of vancomycin followed by degradation-controlled sustained delivery over approximately three weeks. In vitro studies confirmed excellent cytocompatibility, with vancomycin incorporation not affecting cell viability across tested cell lines, even at the highest evaluated concentration (1%). Importantly, antimicrobial assays demonstrated strong, dose-dependent activity against methicillin-resistant *Staphylococcus aureus* (MRSA), including inhibition in both agar diffusion and biofilm-associated titanium implant models, indicating effective activity against implant-related biofilms.

Another study by Censi et al. [40] focused on improving the local delivery of vancomycin through the development of an injectable hydrogel based on vinyl sulfone-functionalized PEG-p(HPMAm-lac) triblock copolymers and thiolated hyaluronic acid (HA-SH). The system provided sustained antibiotic release for at least 5 days, with kinetics governed by diffusion and network properties of the hydrogel. The release profile could be modulated by adjusting the HA-SH content and degree of thiolation, enabling tunable drug delivery. Moreover, the hydrogel preserved vancomycin stability by preventing degradation during encapsulation and release. Antimicrobial testing demonstrated that vancomycin maintained its inhibitory activity against methicillin-susceptible *S. aureus* (MSSA), confirming that hydrogel incorporation did not compromise antibiotic efficacy.

Boot et al. [41] conducted an in vivo rabbit model study to evaluate vancomycin-releasing hydrogels (DAC^®^ hydrogel) as a local treatment strategy for periprosthetic joint infections (PJI). The experimental model involved implantation of sand-blasted titanium devices and induction of infection using *S. aureus*, with animals allocated to groups receiving vancomycin-loaded hydrogels (2% and 5%), unloaded hydrogel, or alternative non-antibiotic agents including bioactive glass (BAG) and N-acetyl-L-cysteine (NAC). Although the hydrogel itself was not expected to exert antibacterial activity, an initial pilot observation suggested a possible difference between unloaded hydrogel and non-coated controls; however, this was not confirmed in the main study, where no significant differences were observed between these groups across infection-related parameters, indicating no intrinsic pro-infective effect of the material. In contrast, vancomycin-loaded hydrogels significantly reduced infection severity, as reflected by lower histological scores, reduced bacterial burden, and improved systemic inflammatory markers, although complete eradication of infection was not consistently achieved. Interestingly, the 2% formulation performed similarly or slightly better than the 5% group, suggesting that higher antibiotic loading did not necessarily improve efficacy. In contrast, neither BAG nor NAC demonstrated in vivo antimicrobial efficacy despite previously reported in vitro activity. Overall, these findings support hydrogel-based local delivery of vancomycin as an effective strategy for improving outcomes in implant-associated infections, while unloaded or non-antibiotic formulations appear insufficient for reliable infection control.

Boot et al. [42] also evaluated a gentamicin-releasing thermo-responsive poly(N-isopropylacrylamide)-grafted hyaluronic acid (HApN) hydrogel in a sheep model of orthopedic device-related infection (ODRI). The study included both prophylaxis and one-stage treatment cohorts, each with corresponding control and hydrogel-treated groups, all receiving standard systemic perioperative antibiotic prophylaxis. In the intervention arms, the gentamicin-loaded hydrogel was applied locally either during primary implantation (prophylaxis) or during revision surgery (one-stage treatment), whereas control groups received no local hydrogel. Infection was induced using methicillin-susceptible *S. aureus* (MSSA), and animals were subsequently followed for assessment of infection outcomes. Results showcased that application of the hydrogel enabled high local antibiotic concentrations with low systemic exposure and showed superiority over standard perioperative intravenous prophylaxis. In both preventive and therapeutic settings, the material reduced bacterial burden and infection severity without relevant toxicity.

Ter Boo et al. [43] evaluated gentamicin-releasing hydrogels in a rabbit implant-associated infection model, comparing a thermo-responsive hyaluronic acid-based hydrogel (HApN) with a clinically established gentamicin-loaded collagen fleece and an untreated control. Following induction of *S. aureus* infection in a femoral osteosynthesis model, both local delivery systems were applied for infection prophylaxis. Collagen fleece served as a benchmark material for local antibiotic delivery, while the HApN hydrogel enabled injectable application with in situ gelation and distribution over the implant site. Both gentamicin-loaded systems effectively prevented infection development, whereas all untreated animals developed established infection confirmed by microbiological and histological analyses. Overall, the HApN hydrogel demonstrated comparable antimicrobial efficacy to the collagen-based reference system, while offering improved adaptability and coverage of complex surgical sites, supporting its potential as a versatile platform for local antibiotic delivery in implant-associated infections.

Single antibiotic-releasing hydrogels, mainly based on vancomycin and aminoglycosides such as gentamycin, demonstrate effective local antimicrobial activity in PJI models with sustained release and proven in vivo and in vitro (animal models) efficacy. Gentamicin systems show performance comparable to established delivery materials such as collagen fleece. However, their spectrum of activity highlights the need for broader, combination-based hydrogel approaches combining multiple antibiotics.

### 3.3. Combination Antibiotic-Loaded Hydrogels

Although numerous single-antibiotic hydrogel systems have been investigated, most recently developed antibiotic-releasing hydrogels incorporate multiple antimicrobial agents.

Boot et al. [44] investigated the efficacy of a gentamicin- and vancomycin-loaded hyaluronic acid hydrogel in a sheep model of MRSA-related ODRI. Following establishment of a chronic infection model, animals were monitored and subsequently treated with the experimental hydrogel. In vitro analysis of the antibiotic-loaded hydrogel demonstrated a rapid burst release of gentamicin and vancomycin within the first 24 h, during which the majority of the antibiotics were released, followed by a gradual decrease in release rate over the subsequent days up to 14 days. The antibiotic-loaded hydrogel exhibited complete bactericidal activity, with no viable bacteria detected, whereas control groups (PBS and antibiotic-free hydrogel) showed persistent bacterial growth. In vivo, the hydrogel was compared with antibiotic-loaded bone cement (ALBC), demonstrating superior efficacy and achieving complete eradication of infection in all treated animals. This effect has been attributed to high local antibiotic concentrations associated with the initial burst release profile, combined with material biodegradability, which eliminates the need for surgical removal. In contrast to ALBC, hydrogels avoid prolonged subinhibitory antibiotic exposure, which may contribute to a reduced risk of antimicrobial resistance. Additional advantages include minimal systemic antibiotic exposure, broad antibiotic loading capacity, and the possibility of repeated intraoperative application in staged revision procedures.

Romano et al. [8] conducted a clinical study evaluating the application of antibiotic-loaded hydrogels in 380 patients undergoing primary or revision total hip or knee arthroplasty with cementless or hybrid implants. The aim was to investigate the use of a DAC^®^ hydrogel loaded with antibiotics as an intraoperative prophylactic strategy to reduce postoperative complications, including PJIs. DAC^®^ (Defensive Antibacterial Coating) is a bioresorbable hyaluronic/polylactic acid hydrogel biodegrading within 72 h. Patients were divided into a treatment group receiving the antibiotic-loaded hydrogel and a control group. The DAC^®^ hydrogel was loaded with various antibiotics, most commonly vancomycin (5%), gentamicin (3.2%), a combination of vancomycin (2%) and meropenem (2%), among others. The results showed a lower incidence of postoperative complications in the treatment group compared to controls. No hydrogel-related adverse effects were reported, suggesting a favorable short-term safety profile, although further long-term studies are required to confirm its efficacy in PJI prevention.

Overstreet et al. [45] evaluated a thermo-responsive poly(N-isopropylacrylamide-co-dimethylbutyrolactone acrylate-co-Jeffamine^®^ M-1000 acrylamide) (PNDJ) hydrogel as a local antibiotic delivery system for the prevention of surgical site infections which also includes development of PJIs. The hydrogel was loaded with vancomycin and tobramycin, and both in vitro drug release kinetics and in vivo performance were assessed in a rabbit model. Prior to animal studies, two antibiotic formulations (3% tobramycin/2% vancomycin and 3% tobramycin/1% vancomycin) incorporated into different PNDJ polymer compositions were screened in order to optimize local delivery characteristics. In vivo experiments demonstrated that antimicrobial release from the hydrogel was significantly faster under physiological conditions compared with in vitro settings. In addition, drug delivery exhibited site-dependent variability, indicating non-uniform release kinetics depending on the injection location. Tissue antibiotic concentrations reached levels comparable to reported MBEC thresholds and were maintained for approximately 24–72 h.

Capuano et al. [10] compared a reduced one-stage revision strategy using an antibiotic-loaded DAC^®^ hydrogel coating with conventional two-stage revision for the treatment of PJIs. The study included 44 patients equally allocated to either treatment group. In the one-stage revision group, the DAC^®^ hydrogel was loaded with 5% vancomycin alone (14 patients) or a combination of 5% vancomycin and 5% meropenem (8 patients). Both groups included five hip and seventeen knee PJI revisions. Clinical outcomes were comparable between the two treatment strategies, with infection recurrence observed in two patients treated with the DAC hydrogel and three patients undergoing two-stage revision. Functional outcomes, assessed using the Harris Hip Score and Knee Society Score, were also similar between groups. Notably, patients treated with the antibiotic-loaded hydrogel experienced shorter hospitalization and a reduced duration of systemic antibiotic therapy, while no hydrogel-related adverse events were reported. These findings suggest that antibiotic-loaded DAC^®^ hydrogel may facilitate less invasive one-stage revision without compromising infection control or functional outcomes.

De Meo et al. [46] evaluated the efficacy of an antibiotic-loaded DAC^®^ hydrogel in preventing postoperative infections following aseptic cementless revision total hip arthroplasty. Thirty-four patients were randomized into two groups: one received standard systemic cefazolin prophylaxis alone, while the intervention group additionally received a DAC^®^ hydrogel applied to the implant surface and loaded with antibiotics. Most patients received gentamicin (200 mg), whereas four patients received a combination of gentamicin (200 mg) and vancomycin (250 mg). During a minimum follow-up of six months, no PJIs were observed in the hydrogel-treated group, compared with six infections in the control group. Importantly, the use of the antibiotic-loaded hydrogel did not adversely affect implant osseointegration or functional outcomes, and no treatment-related adverse events were reported.

Another study by De Meo et al. [47] evaluated an enhanced debridement, antibiotics, and implant retention (DAIR) protocol using an antibiotic-loaded DAC^®^ hydrogel (DACRI) and compared it with the established DAPRI approach utilizing calcium sulfate beads. The study included 16 patients with acute PJIs (eight knee, one shoulder and seven hip prostheses infections). The DAC hydrogel was loaded with pathogen-specific antibiotics selected according to microbiological culture and susceptibility testing, providing local drug release for up to 72 h. Depending on the infecting organism, the hydrogel contained vancomycin alone, vancomycin combined with gentamicin or meropenem, while one patient with Candida albicans PJI received fluconazole-loaded hydrogel. Clinical outcomes demonstrated that DACRI was non-inferior to the established DAPRI protocol in terms of infection control, supporting the use of antibiotic-loaded hydrogels as an effective adjunct to DAIR procedures.

Zagra et al. [9] conducted a study evaluating the addition of antibiotic-loaded hydrogels to a standard cementless two-stage revision protocol for the treatment of PJIs. A total of 54 patients with PJIs were included and divided into two groups, with the intervention group receiving additional treatment with an antibiotic-loaded DAC^®^ hydrogel. The two-stage revision procedure involved removal of the infected implant, extensive debridement, and placement of an antibiotic-loaded spacer. Following a 4–6-week course of systemic antibiotic therapy, patients underwent reimplantation after resolution of clinical and laboratory signs of infection. During the second-stage surgery, the antibiotic-loaded DAC^®^ hydrogel was applied intraoperatively as a coating directly onto the new implant components prior to implantation. The hydrogel was loaded with pathogen-targeted antibiotics, most commonly vancomycin, either alone or in combination with other agents such as meropenem or rifampicin. During follow-up, no adverse effects related to hydrogel application were observed, and no cases of focal osteolysis or implant loosening were reported. No recurrent infections occurred in the hydrogel-treated group, whereas four recurrences were observed in the control group. Overall, the addition of DAC^®^ hydrogel was associated with improved infection control and shorter hospitalization compared with standard treatment alone.

Regarding ongoing clinical investigations, Boyer et al. [48] initiated the SINBIOSE-H trial, a prospective, randomized, open-label study with blinded endpoint assessment (PROBE design) enrolling patients with chronic PJIs. The trial was initiated in 2022 and is scheduled for completion in 2027. It includes 440 patients and aims to compare the effectiveness of a one-stage revision strategy supplemented with antibiotic-loaded DAC^®^ hydrogel with the standard two-stage revision approach. Patients will be followed for up to 24 months to evaluate clinical outcomes and infection control.

Overall, current studies indicate that antibiotic-loaded hydrogels represent a promising and effective approach for the management of implant-related complications, including PJIs. Whether applied as a prophylactic strategy following surgical procedures or as an adjunct to standard treatment protocols, these modified hydrogels have demonstrated favorable therapeutic effects, good biocompatibility, and a low risk of adverse reactions. Several clinical studies have supported their safety and potential effectiveness, suggesting that antibiotic-loaded hydrogels may serve as an additional treatment option in the management of implant-associated infections. However, most available in vivo studies have included relatively short follow-up periods, commonly limited to approximately 6 months. Further investigations with longer observation times are required to better evaluate their long-term efficacy and clinical applicability. Recent studies, such as the clinical trial by Boyer et al. incorporating a 24-month follow-up period, may provide valuable evidence supporting the future implementation of these systems in PJI treatment. We have concluded all the gathered studies regarding antibiotic-loaded hydrogels in a summarizing table—Table 2.

## 4. Emerging Hydrogel-Based Strategies for the Treatment of Periprosthetic Joint Infections

Beyond conventional antibiotic-loaded hydrogels discussed in the previous section, increasing attention has recently been directed toward hydrogel systems incorporating alternative antimicrobial agents, including bacteriophages, nanoparticles, and anti-biofilm enzymes. Although these approaches remain considerably less investigated and are at an earlier stage of development than antibiotic-based hydrogels, they offer several unique mechanisms for combating biofilm-associated infections. In addition, this section discusses smart hydrogels, which are capable of responding to specific environmental stimuli and enabling controlled, on-demand therapeutic release. Collectively, these innovative hydrogel platforms represent promising next-generation strategies for the treatment of biofilm-associated periprosthetic joint infections.

An additional emerging direction is the integration of artificial intelligence (AI) and machine learning (ML) into the development and optimization of advanced hydrogel-based therapies. ML approaches have been explored for optimizing hydrogel formulations and drug-release profiles, while other studies have investigated their ability to predict the antimicrobial efficacy of gel formulations against biofilms [49,50]. AI and ML are also increasingly being investigated in PJI, particularly for diagnosis, risk prediction, prognosis, and treatment optimization [51]. Of particular relevance, Bayat et al. [52] recently combined ML-assisted selection of synergistic bacteriophage combinations with an injectable hydrogel for localized treatment of implant-associated infection. The phage-loaded hydrogel achieved a 5-log reduction in bacterial burden in a biofilm model and improved survival in a murine implant infection model. Although the direct application of AI/ML to hydrogel-based PJI therapy remains limited, these findings highlight the potential of combining computational approaches with advanced biomaterials to optimize antimicrobial combinations, hydrogel formulations, and controlled-release strategies. Such integration may represent an important future direction for the development of more targeted and effective therapies against biofilm-associated PJI.

### 4.1. Bacteriophage-Loaded Hydrogel Systems for Targeted Antibacterial Therapy in PJI

**Figure 2:**
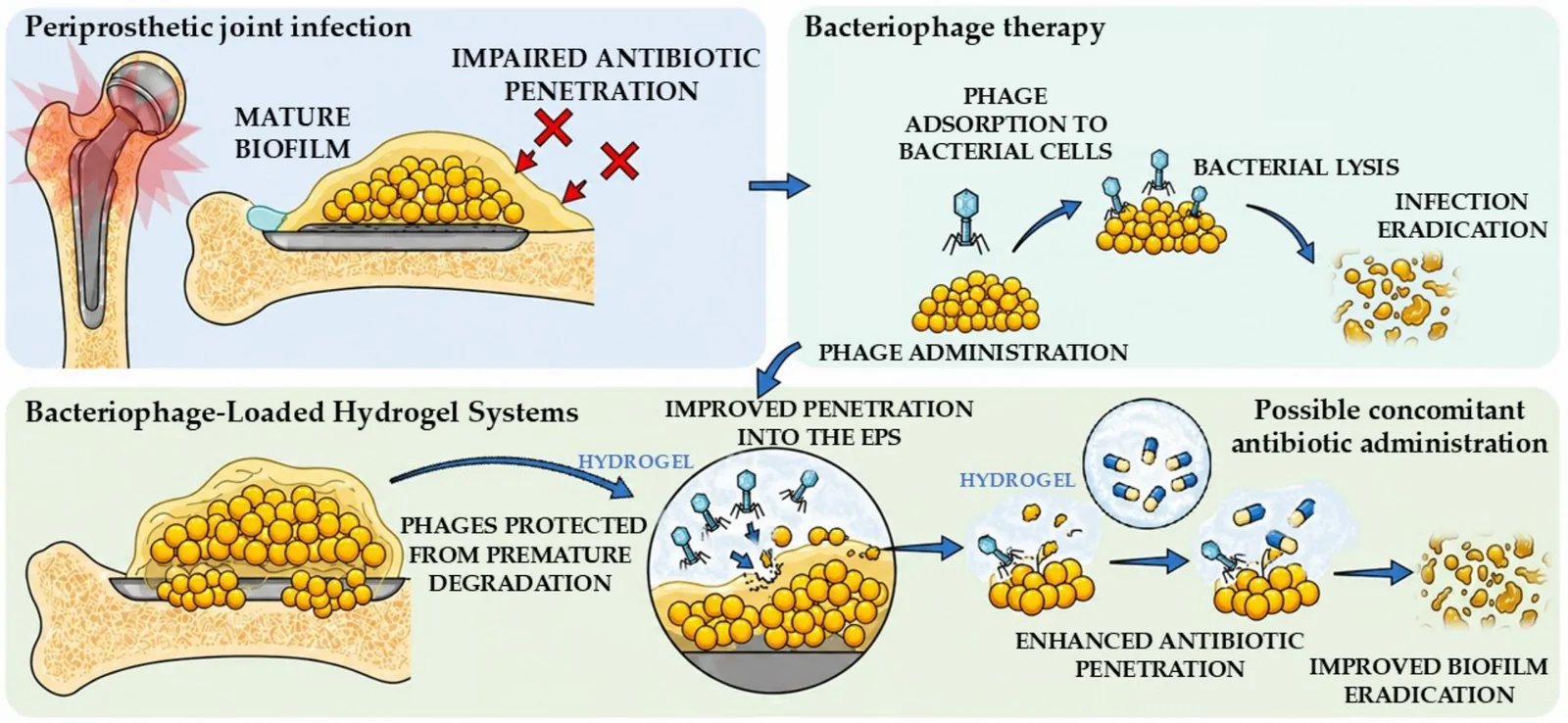
Schematic overview of bacteriophage therapy and bacteriophage-loaded hydrogel systems for the treatment of biofilm-associated periprosthetic joint infections. While mature biofilms limit antibiotic penetration, bacteriophages specifically infect and lyse bacterial cells, disrupting the biofilm structure. Incorporation of bacteriophages into hydrogels enables localized and sustained delivery, enhances phage penetration into the biofilm, and allows combined antibiotic administration, ultimately improving biofilm eradication.

Bacteriophage-loaded hydrogels have emerged as a promising and innovative strategy for the management of PJI, particularly in cases complicated by multidrug-resistant bacteria and mature biofilm formation. PJI remains challenging to treat due to the protective nature of biofilms on implant surfaces, which significantly reduce antibiotic penetration and promote bacterial persistence. In this context, bacteriophages offer a highly specific and self-amplifying antibacterial approach. The rationale for bacteriophage therapy in PJI is based on the natural ability of phages to selectively infect and lyse bacterial cells, particularly Gram-positive pathogens such as *S. aureus* and *Staphylococcus epidermidis*, which are the most common causative agents of PJI. Following adsorption to the bacterial surface, phages replicate intracellularly and induce bacterial lysis, releasing progeny virions that can further propagate the antibacterial effect within the infected microenvironment. This self-amplifying mechanism enables sustained antibacterial activity, especially within biofilm-associated infections where bacterial density is high [53,54,55]. The basic mechanisms of bacteriophage action are presented in Figure 2.

Hydrogels serve as effective delivery platforms for bacteriophages by providing a protective, localized environment that enhances phage stability and activity. Encapsulation within hydrogel matrices protects phages from premature degradation due to physiological conditions, enzymatic activity, and immune clearance, while enabling sustained retention and controlled release at peri-implant sites. Bacteriophage-loaded hydrogels have demonstrated anti-biofilm activity through improved penetration into the EPS matrix and reduction in bacterial burden, with phage-derived depolymerases further enhancing biofilm disruption. Synergistic effects with antibiotics have also been reported, where phage-mediated biofilm breakdown improves antibiotic penetration and reduces the emergence of resistant subpopulations [56].

However, clinical translation in PJI remains limited by variability in phage stability across hydrogel systems, the absence of standardized manufacturing protocols, and regulatory challenges associated with phage-based therapeutics, as well as potential immune neutralization that may reduce long-term efficacy [57,58].

Clinical evidence in orthopedic infections remains limited, with most studies currently restricted to in vitro experiments or animal models. The summaries of the studies listed below are presented in Table 3.

In the study by Kaur et al. [59], bacteriophage-loaded HPMC hydrogel coatings (MR-5 phage) demonstrated a clear, time-dependent reduction in bacterial adhesion and tissue burden in a murine PJI model caused by MRSA. On the implanted K-wires, phage-only coatings reduced adherent bacterial counts from approximately ~6 log CFU (peaking in untreated controls at day 3–5) to around ~4 log CFU during the early phase (day 3–5), followed by a further decline to near-clearance levels by day 7–10. In contrast, control groups maintained high bacterial loads, indicating progressive biofilm formation and infection persistence. In the surrounding joint tissue, phage-treated animals showed a reduction of more than ~3 log CFU compared to untreated controls by day 5–7. Specifically, while infection controls maintained ~8 log CFU in tissue, phage-treated groups decreased to approximately ~4–5 log CFU by day 5, with further decline leading to near sterile or undetectable levels by day 10 in some animals. This reduction correlated with detectable phage replication at the infection site, with titers reaching ~6 log PFU/mL early after implantation and persisting until bacterial clearance, confirming active phage amplification in vivo. The strongest antibacterial effect was observed in the phage + linezolid hydrogel group, where bacterial adhesion on the implant dropped more rapidly (to ~3 log CFU by day 5, compared to ~6 log CFU in controls) and tissue bacterial burden decreased by approximately ~4.5 log CFU by day 5–7 relative to untreated animals. In this dual-treatment group, joint tissue became sterile by day 7, and no bacterial growth was detected thereafter, indicating complete eradication of infection at the local site. Overall, the hydrogel-based phage delivery system achieved multi-log reductions in both implant-associated biofilm and surrounding tissue infection, with reductions of ~2–3 log CFU on the implant and ~3–4.5 log CFU in tissue compared to controls, and even complete sterilization in the dual-therapy group at later time points.

Wroe et al. [60] engineered an injectable PEG-4MAL hydrogel capable of encapsulating *Pseudomonas aeruginosa*-specific bacteriophages while preserving their lytic activity after polymerization and release. The system allowed tunable phage release depending on hydrogel crosslinking and degradation profile, enabling controlled local delivery at the infection site. In vitro, phage-loaded hydrogels significantly reduced bacterial growth and biofilm burden, with up to ~17-fold reduction in viable *P. aeruginosa* recovered from biofilm-associated conditions compared to control hydrogels. Importantly, the encapsulated phages remained active against both planktonic bacteria and mature biofilms under flow conditions, demonstrating effective antibiofilm activity in a physiologically relevant microfluidic model. In vivo, using a murine radial segmental defect model mimicking orthopedic infection, bacteriophage-delivering hydrogels achieved a 4.7-fold reduction in bacterial load at 7 days post-implantation compared to phage-free controls, corresponding to approximately 10^3^ CFU per implant in treated groups. Additionally, viable phages were recovered from treated sites, confirming sustained infectivity and replication at the infection locus. The hydrogel system was also cytocompatible with human mesenchymal stromal cells and did not induce overt adverse tissue responses in vivo.

Barros et al. [61] developed an alginate–nanohydroxyapatite (Alg–nanoHA) hydrogel encapsulating lytic bacteriophages as a multifunctional local delivery system to prevent orthopedic implant-associated infections while supporting bone regeneration. The hydrogel achieved a high phage encapsulation efficiency (~91%) without compromising phage viability, structural integrity, or lytic activity, and released approximately 97% of the encapsulated phages within 24 h under physiological conditions. Phage incorporation did not alter the physicochemical properties of the hydrogel and demonstrated excellent short-term stability during storage. In vitro and in vivo biocompatibility studies showed that the phage-loaded hydrogels did not induce adverse inflammatory reactions or impair osteoblast viability, proliferation, morphology, or differentiation. The presence of nanohydroxyapatite significantly enhanced collagen deposition, mineralization, and bone formation in an ex vivo femoral defect model, and these osteogenic properties were preserved after phage incorporation. Against multidrug-resistant *Enterococcus faecalis*, the phage-loaded hydrogels reduced planktonic bacterial populations by approximately 99% and decreased bacterial adhesion to the hydrogel surface by 98% and to surrounding surfaces by 92%. In an ex vivo infected femur model, bacterial counts were reduced by approximately three orders of magnitude, corresponding to 99.6% and 99.9% inhibition after 24 and 48 h, respectively, with complete prevention of bacterial colonization within bone tissue.

Shiue et al. [62] designed two bacteriophage-functionalized alginate hydrogel coatings for orthopedic implants: a surface-conjugated system and a phage-embedded matrix. Both formulations preserved phage viability and exhibited antibacterial activity against *E. coli*, with the surface-conjugated coating showing faster kinetics, reducing bacterial growth by ~84% within 2 h and preventing biofilm formation at 24 h. The embedded system provided more sustained, diffusion-controlled antibacterial action. Importantly, neither configuration supported biofilm development on the material surface. In vitro, both coatings were cytocompatible, while the surface-conjugated phage layer significantly enhanced osteoblast proliferation (+28.8%) and mineralization (+43.1%). Overall, the study demonstrates that phage immobilization strategy critically influences antibacterial efficacy and osteogenic response, with surface-bound phages offering superior early antimicrobial performance and pro-osteogenic effects.

In the case report by Ferry et al. [63], lytic bacteriophages (PP1493 and PP1815) were incorporated into the DAC^®^ hydrogel and applied locally onto an infected knee megaprosthesis during a DAIR procedure for relapsing *S. aureus* prosthetic joint infection. The hydrogel acted as a carrier enabling rapid local release of phages directly at the implant–biofilm interface while maintaining their biological activity. In vitro testing showed fast release of both phages from the DAC^®^ gel, with stable titers for at least ~6 h (around 10^8^–10^9^ PFU/mL), indicating preserved viability within the matrix. Functionally, PP1493 exhibited strong antibacterial activity with near-complete inhibition of bacterial growth even at low MOI, while PP1815 showed dose-dependent activity requiring higher MOI for full suppression. This confirmed effective anti-*S. aureus* and anti-biofilm potential when adequate local concentrations were achieved. Importantly, DAC^®^ hydrogel did not significantly reduce phage activity or infectivity.

Despite considerable advances in bacteriophage-loaded hydrogel systems for orthopedic infection control, their translation to clinical PJI management remains limited. In particular, there is a marked lack of studies evaluating these platforms in true PJI settings involving titanium implant-associated biofilms and clinically relevant surgical interventions such as DAIR or revision arthroplasty, which remain the gold standard in infection management. Although multiple hydrogel-based phage delivery systems have shown promising antibiofilm efficacy in vitro and in simplified implant or bone defect models, most do not replicate the complex pathophysiology of PJI, including mature biofilms on titanium surfaces under physiological loading and host immune pressure. Moreover, studies directly investigating bacteriophage-loaded hydrogels on clinically relevant titanium implant surfaces under strict PJI conditions remain largely absent. This represents a significant translational gap between biomaterial-based phage delivery strategies and clinically representative orthopedic infection models, highlighting a key and underexplored direction for future research in phage-functionalized implant coatings and hydrogel-based antimicrobial systems.

### 4.2. Nanoparticle-Enhanced Hydrogel Systems for the Treatment of PJIs

**Figure 3:**
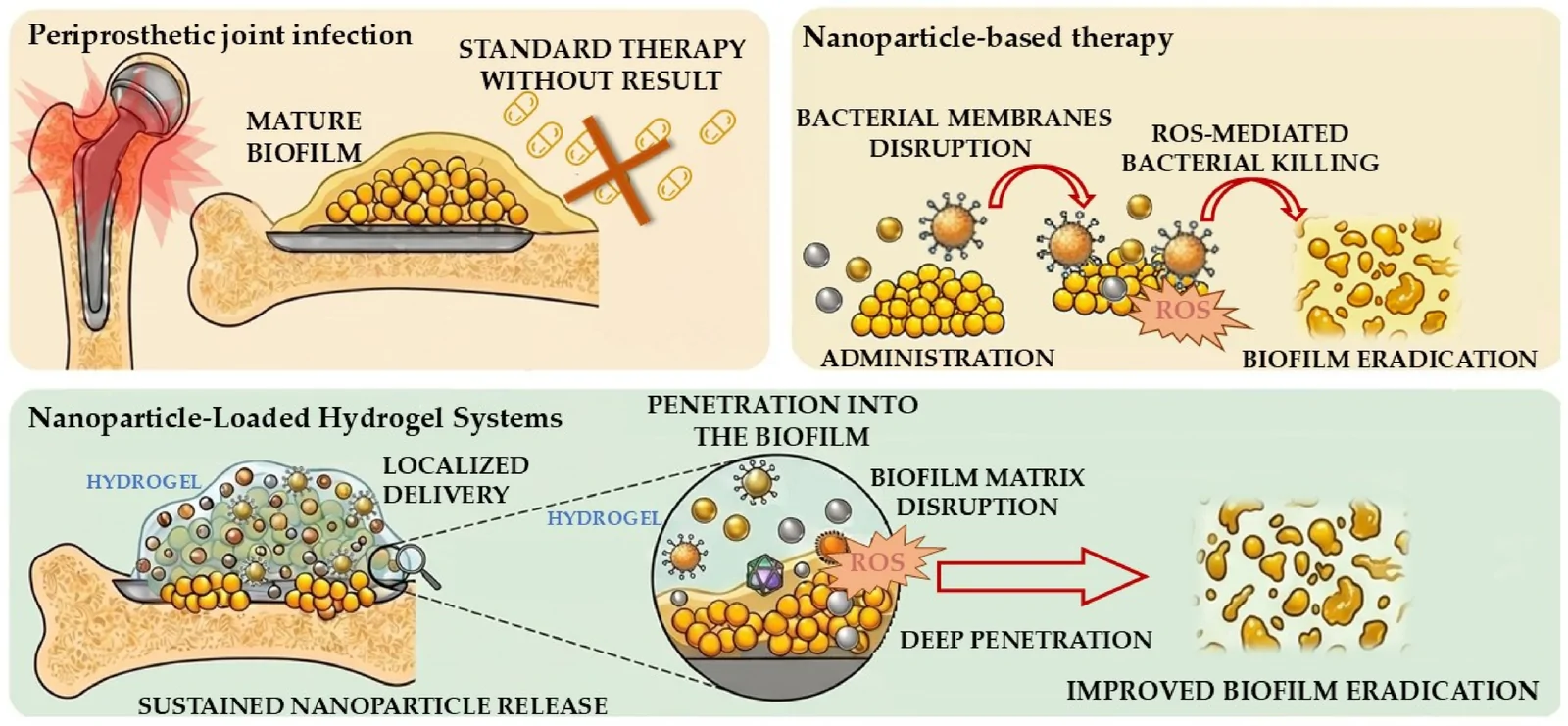
Schematic illustration of nanoparticle-based therapy and nanoparticle-loaded hydrogels for biofilm treatment. Hydrogel-mediated localized and sustained nanoparticle release promotes deep biofilm penetration, biofilm matrix disruption, and ROS-mediated bacterial killing, resulting in improved eradication of persistent biofilms.

Nanoparticles (NPs), typically defined as materials with dimensions ranging from 1 to 100 nm, have attracted considerable interest as multifunctional therapeutic platforms owing to their unique physicochemical properties, including a high surface-to-volume ratio, tunable surface chemistry, and the ability to interact with biological structures at the molecular level. These characteristics enable nanoparticles to penetrate the EPS matrix of bacterial biofilms, disrupt bacterial membranes, generate reactive oxygen species, or serve as carriers for controlled delivery of antimicrobial agents [64,65]. Importantly, in hydrogel-based systems, nanoparticles may therefore contribute to antibacterial therapy through two main mechanisms: they may act as carriers that facilitate the delivery of antimicrobial agents, or they may exert intrinsic antibacterial or antibiofilm activity themselves. The basic mechanism of action of the nanoparticle-loaded hydrogel is shown in Figure 3.

Consequently, nanotechnology has emerged as a promising strategy to overcome the intrinsic antimicrobial tolerance of biofilm-associated infections, including PJIs. The incorporation of nanoparticles into hydrogel matrices represents a logical extension of both technologies, combining the sustained local drug delivery and excellent biocompatibility of hydrogels with the antimicrobial and drug-carrying capabilities of nanomaterials. In this composite system, the hydrogel functions as a localized depot that retains therapeutic agents within the peri-implant environment, whereas nanoparticles improve drug stability, facilitate biofilm penetration, prolong antimicrobial activity [66,67,68]. Depending on the type of nanoparticle used, however, the antibacterial effect may be mediated either by the therapeutic agent carried by the nanoparticle or by the intrinsic properties of the nanoparticle itself. In the former case, nanoparticles primarily serve as delivery platforms, whereas in the latter they act as active antimicrobial components.

Although the application of nanoparticle-enhanced hydrogels specifically in PJI remains at an early preclinical stage, accumulating evidence from orthopedic implant-associated infection models suggests that these multifunctional platforms can simultaneously target bacterial biofilms, improve local antimicrobial delivery, and support the biological processes required for implant integration and bone regeneration. Therefore, nanoparticle-enhanced hydrogels represent a promising next-generation local therapeutic strategy with considerable translational potential for the management of biofilm-associated PJI [69].

Several studies have explored this concept by combining different types of nanoparticles with hydrogel-based delivery systems to achieve enhanced antibiofilm activity against implant-associated pathogens. Among these approaches, photothermal nanomaterials have received particular attention due to their ability to provide localized, non-antibiotic bacterial eradication while preserving surrounding tissues. In such systems, nanoparticles act as active antimicrobial components rather than simply serving as carriers for conventional antibiotics. A brief summary of the cited studies is presented in Table 4.

Wickramasinghe et al. [70] developed a thermoresponsive glycol chitin hydrogel nanocomposite designed to eradicate biofilms associated with orthopedic implant materials. The system combines PEGylated gold nanorods (AuNRs) and D-amino acids (D-AAs), utilizing a dual-action mechanism in which D-AAs disrupt the extracellular polymeric substance of the biofilm, while AuNRs generate localized photothermal effects after activation with 808 nm near-infrared irradiation. The optimized formulation containing 300 ppm AuNRs and 200 mM D-AAs completely eradicated mature *S. aureus* biofilms grown on clinically relevant implant alloys, including Ti-6Al-4V, cobalt-chromium, and tantalum surfaces. In contrast, treatment with D-AAs or photothermal therapy alone resulted only in partial biofilm reduction, with approximately 15–20% residual biofilm remaining. The complete eradication achieved by the combined approach was confirmed using crystal violet staining, colony-forming unit assays, and scanning electron microscopy. Furthermore, the treatment prevented bacterial regrowth and provided effective local heating without detectable damage to surrounding tissue models, demonstrating the potential of nanoparticle-enhanced hydrogels as implant-preserving anti-biofilm therapies. In this formulation, AuNRs primarily function as intrinsically active photothermal antibacterial agents, rather than as carriers of an antimicrobial drug.

While these findings demonstrated the effectiveness of AuNR-based hydrogel systems against mature biofilms on implant materials, further studies were required to evaluate whether this strategy could be translated into clinically relevant PJI models.

Building on these promising in vitro findings, Milbrandt et al. [71] further investigated this hydrogel-based strategy using clinically relevant implant models. Their hydrogel nanocomposite combined D-amino acids with gold nanorods to achieve sequential biofilm disruption and photothermal bacterial killing. The thermoresponsive hydrogel remained injectable as a solution and transformed into a gel at physiological temperature, enabling sustained release of D-AAs and localized photothermal treatment. When applied to mature *S. aureus* biofilms grown on 3D-printed Ti-6Al-4V implants, the combined treatment resulted in complete biofilm eradication (100%), whereas the standard DAIR procedure achieved only approximately 25% biofilm removal. These results provided further evidence that this hydrogel system could overcome the limitations of conventional implant debridement methods.

Following these in vitro studies, Higuera-Rueda et al. [72] evaluated the clinical potential of this technology in an in vivo rabbit model of periprosthetic joint infection. PhotothermAA gel, containing D-amino acids and gold nanoparticles, was tested as an adjunctive therapy combined with DAIR. Rabbits with titanium implants infected with bioluminescent *S. aureus* received DAIR alone or DAIR supplemented with PhotothermAA gel after two weeks of biofilm maturation. In the combined treatment group, implants were coated with the hydrogel, incubated for 2 h, and exposed to an 808 nm laser for 10 min, followed by antibiotic administration. In this case, the gold nanoparticles served primarily as photothermal therapeutic agents, providing direct antibacterial activity following near-infrared irradiation, rather than functioning solely as carriers for the subsequently administered antibiotic. The addition of PhotothermAA significantly reduced implant biofilm coverage compared with DAIR alone (1.8% vs. 81.0%, *p* < 0.0001) and resulted in a greater reduction in bacterial burden in surrounding tissues (5.6-log CFU reduction). No treatment-related tissue necrosis was observed, indicating a favorable safety profile. These findings demonstrate the progression of this hydrogel-based technology from in vitro biofilm eradication studies toward a promising implant-preserving approach.

Beyond photothermal approaches, other nanoparticle-hydrogel systems have been designed to provide multifunctional protection against implant infection by simultaneously targeting bacteria, modulating inflammation, and supporting implant integration. In these systems, nanoparticles may similarly provide intrinsic antibacterial activity, or they may function primarily as nanoscale carriers for therapeutic molecules.

Ding et al. [73] developed a multifunctional nanocomposite hydrogel coating for titanium implants to combat biofilm-associated infections and excessive inflammatory responses. The system consisted of a Schiff-base crosslinked hydrogel composed of aldehyde-modified hyaluronic acid and gelatin, incorporating ZIF-90-Bi-CeO_2_ nanoparticles. The hydrogel coating was chemically anchored to the titanium surface through polydopamine-mediated adhesion, providing stable implant modification. The antibacterial activity resulted from the synergistic effects of the incorporated nanoparticles: bismuth nanoparticles generated photothermal heating, zinc ions provided antibacterial activity, while CeO_2_ nanoparticles exhibited antioxidant properties, reducing oxidative stress in the infected microenvironment. Thus, in this system, the nanoparticles contribute directly to the therapeutic effect through their intrinsic physicochemical and biological properties rather than functioning simply as carriers for conventional antimicrobial agents. In a rat implant-associated infection model, the hydrogel demonstrated effective biofilm removal, modulation of inflammatory responses, and improved osseointegration. This study highlights the potential of nanoparticle-loaded hydrogel coatings as multifunctional platforms that combine antibacterial, anti-inflammatory, and bone-regenerative properties for the treatment of implant-associated infections, including PJI.

In addition to inorganic nanoparticles, nanoscale drug delivery systems have also been incorporated into hydrogel-based implant coatings to achieve controlled release of bioactive molecules with antibacterial and immunomodulatory properties. In contrast to the systems described above, these nanoscale structures primarily function as carriers, with the therapeutic molecule being responsible for the principal antimicrobial or biological activity.

Li et al. [74] developed a multifunctional implant modification strategy based on a 3D-printed porous Ti-6Al-4V titanium alloy scaffold combined with a GelMA–tannic acid (GA) hydrogel loaded with emodin liposomes (EP). In this system, emodin liposomes served as nanoscale drug delivery carriers providing controlled release of emodin, a natural compound with antibacterial and anti-inflammatory properties. Therefore, the liposomes in this formulation primarily serve as drug delivery carriers rather than as intrinsically antibacterial nanoparticles. The composite hydrogel coating enabled sustained release of tannic acid and emodin, while improving implant biocompatibility and regulating the local immune response. The Ti-GA@EP scaffold demonstrated significant antibacterial activity against *S. aureus* and *Escherichia coli*, achieving bacterial inhibition rates of 92% and 88%, respectively, and effectively reduced biofilm formation. In addition, the scaffold promoted macrophage polarization from the pro-inflammatory M1 phenotype toward the regenerative M2 phenotype, reducing inflammatory markers and enhancing osteogenic activity. In vivo studies in a PJI model showed improved infection control and enhanced osseointegration, with a 10.25% increase in bone volume fraction compared with unmodified titanium scaffolds. These findings indicate that emodin liposome-loaded hydrogel coatings represent a promising implant-preserving approach by combining antibacterial, immunomodulatory and bone-regenerative functions for the treatment of PJIs.

Alongside nanoparticle carriers for therapeutic molecules, metal-based nanomaterials remain an important research direction due to their intrinsic antimicrobial activity and ability to directly interfere with bacterial survival mechanisms. Among these materials, silver-based nanoparticles are particularly attractive because of their broad-spectrum antibacterial properties and established activity against antibiotic-resistant pathogens. Unlike nanoparticle-based drug delivery systems, silver nanoparticles can exert antibacterial effects directly, without requiring the incorporation of a conventional antibiotic.

Du et al. [75] developed an injectable polyethylene glycol (PEG) hydrogel loaded with Ångstrom-scale silver particles (Gel-AgÅPs) for the treatment of orthopedic implant-associated infections. In a mouse femoral fracture infection model, Gel-AgÅPs significantly reduced bacterial colonization compared with the untreated infected group. After 7 days of treatment, the hydrogel markedly decreased viable bacterial counts recovered from the femur surface, surrounding muscle tissue, and fixation device, while infected controls treated with Gel-blank showed persistent bacterial growth. At 3 weeks post-infection, Gel-AgÅPs-treated animals remained almost sterile, confirming sustained antibacterial activity. The authors demonstrated that the antimicrobial effect resulted from the incorporation of AgÅPs into the PEG hydrogel matrix, enabling localized and prolonged silver particle release directly at the infection site. The system effectively eliminated *E. coli* infection and reduced infection-associated inflammation, highlighting its potential strategy for preventing and treating orthopedic implant-related infections.

Collectively, these studies demonstrate the versatility of nanoparticle-enhanced hydrogels as multifunctional platforms for PJI management. The reviewed systems highlight two main roles of nanoparticles: serving as carriers for controlled drug delivery or providing intrinsic antimicrobial activity. Some systems combine both mechanisms, potentially enhancing their therapeutic effects. Depending on the incorporated nanomaterial, these systems can provide direct antibacterial activity, biofilm disruption, controlled antimicrobial delivery, modulation of inflammatory responses, and support of implant integration. Although most approaches remain in the preclinical stage, the combination of nanotechnology and hydrogel-based local delivery represents a promising strategy to overcome the limitations of conventional antibiotic therapy and implant revision procedures in biofilm-associated PJI.

### 4.3. Anti-Biofilm Enzyme-Functionalized Hydrogels for Biofilm Disruption in PJI

As mentioned before, PJIs are characterized by the formation of bacterial biofilms on implant surfaces, where microorganisms are embedded in a protective extracellular matrix that limits antibiotic penetration and contributes to persistent infection. Because of this biofilm-associated tolerance, increasing attention has shifted toward enzyme-based, local anti-biofilm strategies delivered via biomaterial carriers such as hydrogels [76]. Among the most relevant enzymatic approaches, DNase I and proteases (e.g., Proteinase K, trypsin) act by degrading structural elements of the extracellular polymeric substance, including extracellular DNA and proteins, which directly destabilizes the biofilm scaffold and increases bacterial susceptibility to antibiotics. In contrast, Dispersin B targets polysaccharide intercellular adhesin (PNAG), while lysostaphin provides direct bactericidal activity against *staphylococci* by cleaving cell wall cross-bridges, making it particularly effective against MRSA-associated biofilms. In addition to matrix-targeting enzymes, quorum quenching strategies such as lactonases interfere with N-acyl-homoserine lactone (AHL)-mediated quorum sensing in Gram-negative bacteria, thereby inhibiting biofilm maturation and virulence regulation rather than directly disrupting the extracellular matrix [77]. Collectively, these enzymatic strategies represent complementary mechanisms that can be integrated into hydrogel-based delivery systems to enhance outcomes in implant-associated infections.

The recalcitrant nature of PJI has increasingly shifted therapeutic strategies toward prevention of biofilm establishment rather than eradication of mature, structurally complex biofilms. In this context, eDNA represents a critical structural and functional component of the biofilm EPS, contributing to scaffold integrity, intercellular cohesion, and restricted antimicrobial penetration. DNase I is a nonspecific endonuclease that hydrolyzes phosphodiester bonds within DNA molecules, resulting in enzymatic degradation of eDNA and subsequent destabilization of the biofilm matrix architecture [78]. This mechanism leads to increased biofilm porosity, disruption of structural cohesion, and enhanced detachment of bacterial aggregates, thereby shifting the microbial phenotype from a sessile, biofilm-associated state toward a more planktonic and therapeutically susceptible form [79]. Importantly, DNase I does not exert direct bactericidal activity; instead, it functions as a matrix-targeting adjuvant that enhances antimicrobial accessibility and efficacy. Preclinical evidence has demonstrated its activity against biofilms formed by major PJI-associated pathogens, including *S. aureus*, *Staphylococcus epidermidis*, and *P. aeruginosa*, supporting its characterization as a broad-spectrum antibiofilm agent [80]. Among currently investigated enzymatic strategies, DNase I is considered one of the most promising candidates for incorporation into local delivery platforms, particularly hydrogel-based systems, due to its well-characterized mechanism of action, demonstrated biofilm-disruptive capacity, and favorable translational profile. In the context of PJI, hydrogel-mediated local delivery of DNase I during perioperative application, debridement, or implant revision represents a rational adjunctive approach aimed at preventing early biofilm maturation and potentiating antibiotic activity. By enzymatically dismantling nascent eDNA-dependent biofilm scaffolds, DNase I significantly enhances antimicrobial penetration and may enable reduced systemic antibiotic exposure while maintaining therapeutic efficacy. Consequently, DNase I is currently regarded as one of the most promising enzymatic agents for hydrogel-based anti-biofilm strategies in the prevention and adjunctive treatment of PJI [78,79,80].

Li et al. [81] investigated a DNase I and liposomal vancomycin-loaded thermosensitive PLGA–PEG–PLGA hydrogel for the treatment of fracture-related infection, demonstrating the key role of DNase I as a biofilm-disrupting enzyme targeting extracellular DNA (eDNA), a fundamental structural component of the MRSA biofilm matrix responsible for scaffold stability, surface adhesion, and limited antibiotic penetration. Within the hydrogel system, DNase I exhibited a rapid burst release, with approximately 77.2% released within 72 h, enabling early degradation of the biofilm during its initial formation phase. Although DNase I alone did not reduce bacterial CFU, it significantly destabilized and dispersed the biofilm structure, as confirmed by reduced crystal violet staining and microscopic evidence of loss of compact biofilm architecture. This enzymatic disruption of the extracellular matrix increased exposure of embedded MRSA cells, thereby enhancing the efficacy of subsequently released vancomycin. The study therefore identifies DNase I as a key matrix-targeting adjuvant that converts a structured, antibiotic-tolerant bacterial community into a dispersed and treatment-susceptible state rather than acting as a direct antimicrobial agent.

Lin et al. [82] demonstrated that DNase I plays a key role in overcoming the physical barrier of MRSA biofilms by enzymatically degrading eDNA. When immobilized within a porous AuAgCu nanozyme hydrogel, DNase I facilitated deeper penetration into the biofilm by disrupting the extracellular matrix, thereby exposing bacteria that are typically protected from antimicrobial agents. Although the study was performed in a murine MRSA-infected wound model rather than in PJI, it underscores the translational potential of DNase-based strategies for implant-associated infections, where effective biofilm penetration is a critical limiting factor for successful treatment.

Proteinase K is a broad-spectrum serine protease with high stability across a wide pH range (4–12) and temperatures of 37–60 °C. It hydrolyzes peptide bonds adjacent to aliphatic and aromatic amino acids and exhibits potent anti-biofilm activity by inhibiting *S. aureus* adhesion and dispersing both early and mature biofilms. Furthermore, Proteinase K acts synergistically with antibiotics, significantly enhancing the degradation of preformed biofilms produced by *S. aureus*, *E. coli*, and several other clinically relevant pathogens [83]. Proteinase K has demonstrated proven efficacy in disrupting biofilms at multiple stages of their development, from inhibiting initial bacterial adhesion to dispersing mature biofilm structures. Moreover, by degrading the proteinaceous components of the extracellular matrix, it enhances the penetration and antimicrobial activity of co-administered therapeutic agents [78,84,85]. Despite the well-established antibiofilm activity of Proteinase K and its synergistic effects with antibiotics, its incorporation into hydrogel-based local delivery systems remains largely unexplored. Proteinase K as a broad-spectrum serine protease has strong anti-biofilm activity; however, its application in hydrogel systems strongly depends on the material used. In protein-based hydrogels (e.g., gelatin, collagen, fibrin), it may cause premature scaffold degradation by cleaving peptide bonds, leading to loss of structural stability. Therefore, it is mainly suitable for synthetic or protease-resistant hydrogels (such as PEG-, PVA-, or PLGA-based systems), which lack susceptible protein substrates. To ensure controlled activity, Proteinase K is typically immobilized or encapsulated so that its action is focused on the biofilm matrix rather than the carrier. Under these conditions, it effectively degrades protein components of the extracellular matrix, enhances biofilm disruption, and improves antibiotic penetration [86].

Trypsin has been shown to effectively disrupt protein-mediated bacterial aggregation and enhance bacterial dispersal in infection-relevant environments, including synovial fluid. This is particularly relevant in orthopedic infections, where protein-rich fluids promote bacterial clustering and reduce antimicrobial accessibility. In biofilm models, trypsin applied in a “trypsin shaving” approach can partially degrade proteinaceous components of EPS, thereby improving access to biofilm-associated bacteria on clinically relevant implant materials such as hydroxyapatite and titanium. However, this enzymatic treatment alone does not lead to significant biofilm eradication. Importantly, a synergistic effect is observed only when trypsin is combined with antibiotics, indicating that proteolytic degradation of the protein-rich matrix enhances antimicrobial penetration and efficacy. Overall, these findings suggest that in the context of PJI, trypsin may serve as adjunctive agents that weaken the protein scaffold of the biofilm and facilitate antibiotic action, although their standalone antibiofilm activity remains limited [87,88]. Trypsin has been successfully incorporated into hydrogel systems as a means of achieving localized and controlled proteolytic activity. In wound healing applications, trypsin-loaded hydrogels have been investigated as platforms that maintain enzyme stability while enabling sustained release at the wound site. This approach allows trypsin to act directly on proteinaceous debris within the wound environment, supporting debridement and improving conditions for tissue repair.

In study by Hu et al. [89] trypsin was incorporated into an injectable, thermosensitive double-crosslinked hydrogel based on quaternized chitosan (QCS), α,β-glycerophosphate (physical crosslinking), and genipin (chemical crosslinking). This system enabled in situ gelation at body temperature and sustained enzyme release directly at the wound site. The study targeted mixed and drug-resistant biofilms formed by clinically relevant pathogens, including *S. aureus*, MRSA, *P. aeruginosa*, and multidrug-resistant *P. aeruginosa* (MDR-Pa). In vitro, the trypsin-loaded hydrogel disrupted biofilms by degrading EPS, leading to strong biofilm dispersion and significantly improved antimicrobial efficacy compared to hydrogel alone or antibiotics. Importantly, trypsin alone was non-bactericidal but strongly enhanced bacterial exposure to antimicrobial action. In vivo, the system was tested in a diabetic rat full-thickness wound model infected with a mature mixed biofilm composed of *S. aureus*, MRSA, and *P. aeruginosa*. The trypsin-loaded hydrogel significantly accelerated biofilm clearance, reduced inflammation, and improved healing outcomes, including faster re-epithelialization, increased collagen deposition, and enhanced angiogenesis compared to controls.

An interesting aspect of trypsin is its role as a biological switch controlling drug release. In the study by Bourgat et al. [90], an enzyme-responsive alginate-based nanogel system was developed for controlled antibiotic delivery. The hydrogel is composed of ionotropically crosslinked alginate and poly-L-lysine (PLL), with ciprofloxacin covalently attached to PLL via an enzymatically cleavable peptide linker. PLL serves both as a structural crosslinker stabilizing the alginate network and as a protease-sensitive component. Trypsin, used as a model inflammatory protease, specifically hydrolyzes peptide bonds in PLL, leading to progressive degradation of the polymer chains. This cleavage disrupts electrostatic interactions between PLL and alginate, resulting in destabilization and gradual disassembly of the nanogel structure, which subsequently triggers ciprofloxacin release. The system was evaluated in vitro against *S. aureus*, a clinically relevant Gram-positive bacterium commonly associated with implant-related infections. In the presence of trypsin, particle size increased due to network loosening followed by structural breakdown, accompanied by a clear, concentration-dependent increase in ciprofloxacin release.

Dispersin B is a PNAG-specific glycosidase that enzymatically hydrolyses β-1,6-linked N-acetylglucosamine polymers, a major structural component of staphylococcal biofilm extracellular matrix. In the context of PJI, where PNAG-producing *S. aureus* strains are key pathogens, degradation of PNAG leads to loss of matrix integrity, reduced intercellular adhesion, and biofilm destabilization. This results in increased biofilm porosity and detachment of bacterial cells from the implant surface, without directly exerting bactericidal activity. Consequently, dispersin B converts sessile biofilm-associated bacteria into a more planktonic phenotype, thereby significantly enhancing susceptibility to antimicrobial agents. Its efficacy is strain-dependent, reflecting variability in PNAG contribution to biofilm architecture, with reduced activity in PNAG-independent, protein- or eDNA-dominant matrices [91]. In PJI-relevant models, dispersin B is therefore one of the best characterized as a biofilm matrix-disrupting adjuvant that potentiates antibiotic penetration and activity rather than a primary antimicrobial agent [92,93]. However, its application in hydrogels has not yet been thoroughly investigated, which may represent an interesting direction for future research. Only in study by Hagen et al. [94], Dispersin B was incorporated into a degradable VetriGel hydrogel alongside amikacin to evaluate its in vitro elution characteristics. Dispersin B, an enzyme that degrades the extracellular matrix of bacterial biofilms, exhibited a rapid initial release within the first 24 h, followed by a gradual decline over the 10-day observation period. Overall, only approximately 7% of the total loaded dose was released, indicating limited elution efficiency from the hydrogel matrix. There was no significant difference in total cumulative release between Dispersin B alone and when combined with amikacin, suggesting that the antibiotic did not meaningfully affect its overall elution profile, although some time-dependent variations were observed.

Lysostaphin is a zinc-dependent metallo-endopeptidase produced by *S. simulans* that exhibits highly specific antimicrobial activity against staphylococcal species. Its specificity is mediated by a distinct cell wall-binding domain that recognizes *S. aureus* peptidoglycan, while its catalytic domain cleaves the pentaglycine interpeptide cross-bridges, which are essential for maintaining the structural integrity of the bacterial cell wall. Enzymatic disruption of these cross-links results in rapid osmotic instability and bacterial cell lysis [95]. Unlike conventional small-molecule antibiotics, lysostaphin exerts bactericidal activity independent of bacterial metabolic activity, enabling effective killing of both actively dividing and metabolically quiescent populations, including bacteria embedded within biofilms [96]. Consequently, lysostaphin represents a promising therapeutic agent for PJI, particularly those caused by MRSA, where conventional antimicrobial strategies frequently demonstrate limited efficacy due to resistance and biofilm-mediated protection [97].

The study by Johnson et al. [98] investigates injectable PEG-based hydrogels as a local delivery system for lysostaphin to treat *S. aureus* orthopedic implant infections. Lysostaphin, a bacteriolytic enzyme specific for staphylococci, was encapsulated in a PEG-4MAL hydrogel that adheres to fracture and tissue surfaces and enables sustained, protease-responsive release. The hydrogel preserved enzyme activity and improved stability compared to soluble lysostaphin, while also enhancing antibacterial and antibiofilm efficacy in vitro against multiple *S. aureus* strains, including MRSA and *S. epidermidis*. In a murine femur fracture infection model, lysostaphin-loaded hydrogels completely eradicated infection in bone, surrounding tissue, and implants, outperforming both systemic oxacillin prophylaxis and free lysostaphin, which showed inconsistent or poor bacterial clearance. The system was effective against biofilm-associated bacteria and antibiotic-resistant strains. Cytokine profiling showed restoration of a sterile-like inflammatory environment, with normalization of pro-inflammatory markers (e.g., IL-1β, IL-6, TNF-related cytokines). Importantly, infection clearance enabled normal fracture healing, confirmed by μCT, histology, and mechanical testing, showing bone regeneration and strength equivalent to uninfected controls. In contrast, untreated infected fractures exhibited persistent infection and impaired healing.

In another study Johnson et al. [96] developed a PEG-4MAL hydrogel system enabling simultaneous antimicrobial activity and bone regeneration by co-delivering lysostaphin and BMP-2. Lysostaphin, a staphylococcal-specific endopeptidase, effectively disrupts *S. aureus* biofilms and eliminates infection, while BMP-2 promotes osteoinduction and new bone formation. In a murine critical-sized radial defect model with *S. aureus* infection, the hydrogel achieved complete bacterial clearance and restored bone regeneration to levels comparable to uninfected controls, both structurally and mechanically. Lysostaphin delivery did not induce excessive local inflammation, and cytokine and immune cell profiles indicated restoration toward a sterile wound environment. These suggest that combined local delivery of antimicrobial enzymes and osteogenic factors represents a promising strategy for managing complex orthopedic infections with bone loss, with potential relevance to PJI, where eradication of biofilm and simultaneous tissue regeneration remain major clinical challenges.

Lactonases (N-acyl-homoserine lactone acylases/hydrolases) are quorum quenching enzymes that catalyze the hydrolysis of N-acyl-homoserine lactone (AHL) signaling molecules, which are key mediators of quorum sensing in Gram-negative bacteria. By enzymatically degrading AHLs, lactonases disrupt intercellular communication pathways that regulate biofilm maturation, virulence factor production, and coordinated bacterial behavior. As a consequence, bacteria are unable to maintain a fully organized biofilm phenotype and exhibit reduced expression of adhesion- and persistence-associated genes, resulting in impaired biofilm development and increased susceptibility to antimicrobial agents. In the context of PJI, where polymicrobial biofilms and quorum sensing-driven virulence contribute to chronic infection and antibiotic tolerance, lactonase-based strategies represent a complementary anti-biofilm approach distinct from matrix-degrading enzymes such as DNase I or proteases. Rather than directly disrupting the EPS, lactonase targets bacterial communication systems, thereby preventing biofilm maturation at an early stage and attenuating pathogenicity. Preclinical studies suggest that quorum quenching enzymes may enhance the efficacy of conventional antibiotics and other anti-biofilm agents when incorporated into local delivery systems such as hydrogels, supporting their potential role as adjunctive therapeutics in implant-associated infections caused by Gram-negative bacteria [99,100].

Overall, enzyme-based strategies delivered via hydrogel systems represent a promising and highly innovative direction in the management of biofilm-associated implant infections. By targeting different levels of biofilm biology—ranging from structural components of the extracellular matrix (eDNA, proteins, polysaccharides) to bacterial cell wall integrity and quorum sensing regulation—these approaches offer a multifaceted means of overcoming the intrinsic tolerance of biofilm-embedded bacteria to conventional antibiotics. Despite strong preclinical evidence demonstrating enhanced biofilm disruption, improved antibiotic penetration, and increased bacterial susceptibility, most of these strategies remain at an early experimental stage. Data are largely limited to in vitro studies and animal models, and their efficacy, safety, stability within biomaterial systems, and translational performance in clinical settings remain insufficiently characterized. Therefore, although enzyme-functionalized hydrogels constitute a novel and mechanistically rational platform for local therapy in periprosthetic joint infection, further systematic research is required. In particular, studies addressing long-term biocompatibility, controlled enzyme release, resistance development, and clinical effectiveness are essential before these approaches can be implemented in routine clinical practice.

### 4.4. Smart Hydrogels—Future Perspectives for PJI Treatment

Smart hydrogels have emerged as an advanced generation of hydrogel-based drug de-livery systems designed to overcome some limitations of conventional hydrogels. While traditional hydrogels can provide localized and sustained release of therapeutic agents, their release profiles are generally predetermined by the physicochemical properties of the material and are not dynamically adjustable after administration. In contrast, smart hydrogels are engineered to respond to specific internal or external stimuli, such as pH, temperature, enzymes, light, or bacterial-associated signals, enabling more precise and temporally controlled drug release [26]. This stimulus-responsive behavior creates the possibility of delivering antimicrobial agents specifically under conditions associated with infection, potentially improving therapeutic efficacy while reducing unnecessary exposure and minimizing the risk of adverse effects [101].

#### 4.4.1. pH-Responsive Loaded Hydrogels

pH-responsive drug-loaded hydrogels represent one of the most promising classes of stimulus-responsive biomaterials for the local treatment of infections. Their therapeutic potential is based on the acidic microenvironment that develops during bacterial infection and inflammation, allowing selective release of therapeutic agents at the site of pathology while minimizing drug release in healthy tissues [102]. Although this approach has not yet been extensively investigated in PJI models, encouraging results have been reported in other infection- and inflammation-related applications. Li et al. [103] developed a pH-responsive injectable self-healing hydrogel formed through dynamic cross-linking of amphoteric chitosan and multi-armed polyethylene glycol and loaded with curcumin-preconditioned mesenchymal stem cell-derived exosomes. The hydro-gel was designed to release its therapeutic cargo under mildly acidic conditions (pH 4.5–6.5), characteristic of chronic diabetic wounds. Both in vitro and in vivo studies demonstrated targeted pH-triggered release, resulting in reduced inflammation, enhanced angiogenesis, and accelerated wound healing in a diabetic rat model. Although these findings originate from a non-PJI application, they highlight the potential of pH-responsive hydrogels as infection-triggered local drug delivery systems that may be translated to the treatment of PJIs.

#### 4.4.2. Enzyme—Responsive Loaded Hydrogels

Enzyme-responsive hydrogels represent another promising class of stimulus-responsive drug delivery systems. Since bacterial pathogens produce a variety of enzymes that are absent or present at negligible levels in healthy tissues, these enzymes may serve as highly specific triggers for local drug release. In the context of PJIs, enzymes produced by staphylococci and other biofilm-forming pathogens could potentially be exploited to achieve bacteria-specific antimicrobial delivery. Abbasi et al. [104] developed a β-lactamase-responsive antibacterial hydrogel incorporating ciprofloxacin-loaded liposomes. Prior to in vivo evaluation in a murine wound infection model, the hydrogel demonstrated enzyme-triggered degradation and on-demand ciprofloxacin release in vitro, while remaining stable under physiological conditions. Both in vitro and in vivo experiments demonstrated complete eradication of *P. aeruginosa* infection, superior wound healing compared with a commercially available silver dressing, excellent cytocompatibility, and no detectable induction of ciprofloxacin resistance under non-triggering conditions. Although, similarly to pH-responsive systems, this hydrogel has not yet been evaluated in PJI models, its bacteria-specific drug release mechanism represents a promising strategy for the future treatment of im-plant-associated biofilm infections.

#### 4.4.3. ROS—Responsive Loaded Hydrogels

ROS represent another promising stimulus for the development of smart hydrogels in the context of PJIs. Together with an acidic pH, increased ROS levels are a characteristic feature of infected and inflamed microenvironments, making them an attractive trigger for infection-responsive drug delivery systems. ROS-responsive loaded hydrogels could potentially enhance PJI treatment by enabling localized and inflammation-triggered therapeutic release while simultaneously modulating the oxidative environment. In other fields, particularly when combined with pH responsiveness, ROS-sensitive hydrogel platforms have demonstrated promising therapeutic potential. Zhu et al. [105] developed a ROS-responsive hydrogel system for the delivery of nanoparticles in periodontitis treatment. Ultra-small Fe-Que nanoparticles were incorporated into a hyaluronic acid-based hydrogel modified with ROS-cleavable bonds, enabling stimulus-responsive behavior. In vitro studies demonstrated strong antibacterial activity, effective ROS scavenging capacity, and immunomodulatory properties through the reduction in pro-inflammatory responses and enhancement of antioxidant activity. Furthermore, in a rat model of periodontitis, the hydrogel effectively reduced inflammation, promoted tissue regeneration, and facilitated alveolar bone repair. Although ROS-responsive hydrogels have not yet been evaluated in PJI models, these systems represent a promising future plat-form for implant-associated infection treatment by combining pathogen/inflammation-triggered drug delivery with simultaneous regulation of the oxidative and immune microenvironment.

Overall, smart loaded hydrogels represent an emerging concept that may offer new possibilities for future PJI treatment. By responding to specific features of the infected microenvironment, such as acidic pH, bacterial enzymes, or elevated ROS levels, these systems could potentially enable more precise and controlled therapeutic delivery. Although their application in PJI has not yet been highly investigated, results from other fields highlight the potential of stimulus-responsive hydrogels as a future strategy for improving localized treatment of implant-associated infections.

## 5. Materials and Methods

In this narrative review, we analyzed studies published from 2016 onwards focusing on the application of hydrogels for local drug delivery in biofilm-associated periprosthetic joint infections (PJIs). The literature search included keywords such as “hydrogel PJI”, “antibiotic-loaded hydrogel”, “local antibiotic delivery”, “biofilm infection”, “antimicrobial hydrogel”, “smart hydrogel”, “phage-loaded hydrogel”, and “anti-biofilm enzyme delivery”. The reviewed studies included in vitro investigations, animal models, and clinical studies evaluating hydrogels as platforms for PJI prevention and treatment. Particular attention was given to antibiotic-loaded hydrogels, hydrogel-based anti-biofilm strategies, phage delivery systems, and stimuli-responsive (“smart”) hydrogels. Studies were selected based on their relevance to PJIs, with emphasis placed on recent advances and emerging therapeutic approaches.

## 6. Conclusions

Hydrogels represent a versatile platform for localized drug delivery in biofilm-associated PJI. Their capacity to provide sustained and spatially confined release of antimicrobial agents at the infection site makes them a potential adjunct to established surgical and systemic antimicrobial strategies. Antibiotic-loaded hydrogels remain the most extensively studied approach and are supported by preclinical evidence and emerging clinical data, particularly in conjunction with revision procedures and debridement, antibiotics, and implant retention. Nevertheless, current evidence remains insufficient to support their use as standalone therapies, and their clinical application should presently be regarded as complementary to appropriate surgical and systemic antimicrobial treatment.

Despite encouraging findings, several barriers continue to limit the clinical translation of hydrogel-based antimicrobial and anti-biofilm systems. Considerable heterogeneity in polymer composition, crosslinking strategies, degradation profiles, drug-loading approaches, and incorporated antimicrobial agents complicates standardization and comparison across studies. Moreover, simplified experimental models may not adequately reproduce the complex PJI microenvironment, where mature biofilms, synovial fluid, inflammatory processes, implant surfaces, tissue interactions, and mechanical loading can influence hydrogel stability and drug-release kinetics. Achieving an appropriate balance between therapeutic persistence, controlled biodegradation, mechanical integrity, and tissue compatibility therefore remains a major challenge. Further limitations include formulation stability, sterilization, manufacturing reproducibility, regulatory requirements, and the limited availability of adequately powered clinical studies with long-term follow-up.

Future development should focus on the rational design of multifunctional systems targeting complementary mechanisms of biofilm persistence. Bacteriophage-loaded hydrogels, nanoparticle-enhanced platforms, and enzyme-functionalized systems may enhance bacterial eradication through targeted antimicrobial activity, improved biofilm penetration, extracellular polymeric substance disruption, or interference with bacterial communication. However, most of these approaches remain at the preclinical stage, emphasizing the need for validation in clinically relevant implant-associated biofilm models and well-characterized in vivo studies. In particular, future research should prioritize models that reproduce mature biofilms on clinically relevant implant materials under physiologically and surgically representative conditions.

“Smart” hydrogels represent another important direction, enabling controlled drug release in response to stimuli such as pH, reactive oxygen species, enzymatic activity, or temperature. The incorporation of multiple therapeutic functions, including antimicrobial, anti-biofilm, tissue-regenerative, or anti-inflammatory activities, may further enhance their clinical relevance. Emerging artificial intelligence and machine learning approaches may additionally facilitate the optimization of hydrogel composition, antimicrobial combinations, and drug-release kinetics, supporting a more rational and data-driven development process.

Future research should prioritize not only antimicrobial efficacy but also the development of reproducible, clinically relevant, safe, and scalable therapeutic platforms. Standardized testing protocols, clinically representative biofilm and implant models, long-term evaluation of degradation and tissue responses, reproducible manufacturing and sterilization procedures, and adequately powered prospective clinical trials will be essential for establishing their clinical value. Extended follow-up will also be required to determine whether the favorable outcomes reported for selected systems translate into durable infection control and improved patient outcomes.

Overall, hydrogel-based drug delivery systems constitute a rapidly evolving field with considerable potential to complement current therapeutic strategies for biofilm-associated PJI. Successful clinical translation will require a shift from isolated proof-of-concept studies toward standardized, multifunctional, mechanistically defined, and clinically validated platforms. Continued interdisciplinary collaboration will be essential to translate advances in hydrogel engineering into reliable therapeutic options capable of improving biofilm eradication and long-term outcomes in patients with PJI.

## Figures and Tables

**Table 1:** Multifunctional classification of hydrogels for biofilm-associated periprosthetic joint infection therapy.

Classification Criterion	Hydrogel Type	Main Characteristics	Advantages in PJIs	Limitations
Polymer origin	Natural (alginate, chitosan, collagen, gelatin, etc.)	Derived from biopolymers (alginate, chitosan, collagen, gelatin, hyaluronic acid, fibrin); ECM-like structure	Excellent biocompatibility, ECM-mimicking structure supporting local tissue regeneration and implant–tissue integration	Low mechanical strength, rapid degradation, batch-to-batch variability
	Synthetic (PEG, PVA, PAAm, etc.)	Chemically defined polymers with precisely known molecular structure and composition	High reproducibility, tunable mechanical properties, precise control of antibiotic loading and sustained release kinetics in infected peri-implant sites	Limited biological activity, often requires biofunctionalization
	Hybrid (PEG–gelatin, PEG–hyaluronic acid, chitosan–PVA)	Combination of natural and synthetic polymers with complementary biological and mechanical properties	Balanced antimicrobial delivery and mechanical stability for long-term local treatment in biofilm-associated infections	More complex synthesis and formulation optimalization
Crosslinking mechanism	Physical	Non-covalent interactions (hydrogen bonds, ionic, hydrophobic interactions, crystallization)	Mild preparation enabling encapsulation of antibiotics, proteins, and bacteriophages without loss of bioactivity; suitable for local delivery in infected tissues, self-healing potential	Lower mechanical stability and less predictable drug release
	Chemical	Covalent crosslinking (photo-crosslinking, Schiff base, click chemistry, enzymatic reactions)	Stable covalent network enabling prolonged antibiotic retention and sustained high local concentrations in the peri-implant space	Potential cytotoxicity
Degradability	Biodegradable (gelatin, hyaluronic acid, etc.)	Hydrolytic or enzymatic degradation into non-toxic products	No removal required; degradation matched to treatment duration	Limited long-term structural support if necessary.
	Non-biodegradable (PVC, PAAm, PU, PHEMA, etc.)	Minimal in vivo degradation	Long-term local antibiotic depot enabling extended suppression of residual biofilm-associated bacteria	Potential bacterial recolonization
Stimuli responsiveness	Conventional	Passive diffusion and matrix degradation	Simple fabrication, passive antibiotic diffusion for baseline local infection control	Limited control over release
	Stimuli-responsive “smart”	Respond to pH, enzymes, ROS, temperature or other stimuli	Infection-triggered antibiotic release in response to pH/ROS/enzymes, enabling biofilm-targeted therapy, reduced site-specific drug delivery, reduced toxicity	More complex manufacturing, not well established in PJIs
Physical form	Injectable	Liquid precursor with in situ gelation	Minimally invasive delivery into peri-implant space, suitable for DAIR	Limited initial mechanical strength
	Performed	Solid or semi-solid construct fabricated before implantation	High structural stability, defined antibiotic-loaded scaffold for localized delivery in large defects requiring structural support	More invasive implantation
	In situ gelling	Gelation triggered by physiological conditions (temperature, pH, ions)	Conformal adaptation to irregular infected cavities with precise localization, minimally invasive	Gelation behavior could depend on unpredictable physiological environment, not well established in PJIs

**Table 2:** Summary of studies evaluating antibiotic-loaded hydrogels for the treatment and prophylaxis of PJIs.

Authors and Year	Study	Antibiotics	Hydrogel	Mechanism of Drug Release	Notable Information	References
Liao et al. (2020)	In vitro study on drug release, antimicrobial activity against *S. aureus*, biocompatibility and antibiofilm activity of the loaded-hydrogel	Vancomycin (0.01–1%)	oxi-HA/ADH hydrogel	Diffusion-controlled	Initial burst release followed by sustained vancomycin delivery for ~3 weeks, with excellent cytocompatibility and effective dose-dependent activity against MRSA, including biofilm-associated implant models.	[39]
Censi et al. (2019)	In vitro study on Vancomycin release from the hydrogel, degradation of the released antibiotic, activity against MSSA	Vancomycin	PEG-p(HPMAm-lac1-2)/thiolated hyaluronic acid hydrogel	Diffusion-controlled + ionic dissociation	Loaded-hydrogel sustained vancomycin release for ≥5 days with tunable release kinetics depending on HA-SH content, while preserving antibiotic stability and antimicrobial activity against MSSA	[40]
Boot et al. (2020)	In vivo study on New Zealand White rabbits using an implant-associated infection model with sand-blasted titanium implants and *S. aureus*, comparing vancomycin-loaded hydrogel prophylaxis with bioactive glass (BAG) and N-acetyl-L-cysteine (NAC).	Vancomycin (2% and 5%)	DAC^®^ hydrogel	Diffusion-controlled	Vancomycin-loaded hydrogels reduced infection severity and bacterial load in a *S. aureus* titanium implant model; higher loading (5%) offered no advantage, and BAG/NAC were ineffective in vivo	[41]
Boot et al. (2022)	In vivo ovine chronic ODRI (8 weeks) model with intramedullary tibial nail implantation and MSSA infection, standard prophylaxis, one-stage revision surgery and hydrogel application	Gentamycin (200 mg per sheep)	poly(N-isopropylacrylamide) grafted hyaluronic acid hydrogel	Diffusion-controlled (initial burst release)	High local antibiotic concentrations with low systemic exposure; superior to intravenous prophylaxis and reduced bacterial burden and infection severity in both preventive and therapeutic settings without relevant toxicity.	[42]
Ter Boo et al. (2016)	In vitro antibiotic release study and in vivo New Zealand White rabbit implant-infection model using surgical plating with gentamicin-sensitive *S. aureus* isolated from a patient with knee PJI, comparison with antibiotic-loaded collagen fleece	Gentamycin (1% and 2%)	HApN (hyaluronic acid–pNIPAM hydrogel)	Diffusion-controlled + thermoresponsive in situ gelation (initial burst release)	Gentamicin-loaded HapN hydrogel showed comparable infection prevention efficacy to collagen fleece in a *S. aureus* femoral osteosynthesis model, while offering improved injectability and better coverage of the implant site.	[43]
Boot et al. (2021)	In vitro antibiotic release study combined with an in vivo ovine chronic ODRI model of MRSA infection, comparison with ALBC, assessing systemic antibiotic concentrations, safety and antimicrobial activity	Gentamycin (1%) + Vancomycin (4%)	hyaluronic acid-based hydrogel	Diffusion-controlled + thermoresponsive in situ gelation (initial burst release)	Hydrogel expressed burst release of gentamicin and vancomycin within 24 h followed by sustained release up to 14 days, resulting in complete in vitro bactericidal activity and full infection eradication in vivo, outperforming antibiotic-loaded bone cement (ALBC) while enabling high local exposure with minimal systemic levels and avoiding surgical removal.	[44]
Romano et al. (2016)	Multicenter clinical study including 380 patients undergoing prosthesis surgery evaluating loaded-hydrogel application for reduction in postoperative complications.	Vancomycin (5%), Gentamycin (3.2%), Vancomycin (2%) + Meropenem (2%)	DAC^®^ hydrogel	Chemically controlled—hydrolytic degradation	Lower incidence of postoperative complications in the hydrogel group compared to controls, with no reported material-related adverse effects	[8]
Overstreet et al. (2019)	In vitro drug release kinetics and in vivo study on New Zealand White rabbits with *S. aureus*	Tobramycin (3%) + vancomycin (2%), Tobramycin (3%) + Vancomycin (1%)	poly(N-isopropylacrylamide-co-dimethylbutyrolactone acrylate-co-Jeffamine^®^ M-1000 acrylamide) (PNDJ) hydrogel	Diffusion-controlled + thermoresponsive gelation (sustained release)	Faster antimicrobial release under in vivo physiological conditions compared with in vitro, with site-dependent variability in delivery. Tissue concentrations reached MBEC-comparable levels and were sustained for approximately 24–72 h; study did not involve any implanted materials, more focus on reducing risk of surgical site infections	[45]
Capuano et al. (2018)	Two-center case–control study involving 44 patients, comparing a one-stage procedure with antibiotic-loaded hydrogel to the standard two-stage procedure.	Antibiotics selected according to patient-specific microbiological culture results.	DAC^®^ hydrogel	Chemically controlled—hydrolytic degradation	Comparable infection control and functional outcomes to two-stage revision, with shorter hospitalization and reduced systemic antibiotic therapy, and no hydrogel-related adverse events.	[10]
De Meo et al. (2020)	Retrospective case–control observational study involving 34 patients, comparing standard prophylaxis alone with standard prophylaxis supplemented by antibiotic-loaded hydrogel application.	Gentamycin (200 mg in 5 mL hydrogel) Gentamycin (200 mg in 5 mL hydrogel) + Vancomycin (250 mg in 5 mL hydrogel)	DAC^®^ hydrogel	Chemically controlled—hydrolytic degradation	No PJIs occurred in the hydrogel-treated group compared with six cases in controls during ≥6 months follow-up; no adverse effects on osseointegration or functional outcomes were observed.	[46]
De Meo et al. (2023)	Clinical study involving 16 PJI patients undergoing modified DAIR procedures	Antibiotics selected according to patient-specific microbiological culture results.	DAC^®^ hydrogel	Chemically controlled—hydrolytic degradation (initial burst release)	DACRI demonstrated non-inferior outcomes compared with DAPRI, with comparable safety and effectiveness in PJI treatment.	[47]
Zagra et al. (2018)	Clinical study involving 54 patients comparing a standard two-stage cementless procedure with additional antibiotic-loaded hydrogel application.	Antibiotics selected according to patient-specific microbiological culture results.	DAC^®^ hydrogel	Chemically controlled—hydrolytic degradation	No hydrogel-related adverse events, osteolysis, or implant loosening were observed. No infection recurrences occurred in the hydrogel group compared with four in controls, with improved infection control and shorter hospitalization.	[9]
Boyer et al. (2025)	Prospective, randomized, open-label study with blinded endpoint assessment involving 440 patients with chronic PJIs, comparing one-stage revision with adjunctive antibiotic-loaded hydrogel application versus standard two-stage revision.	Antibiotics will be selected according to patient-specific needs from agents compatible with the hydrogel	DAC^®^ hydrogel	Chemically controlled—hydrolytic degradation	Currently ongoing (2022–2027), will include longer 24 month follow-up observation of the patients	[48]

**Table 3:** Summary of experimental outcomes of bacteriophage-loaded hydrogel systems in orthopedic infection models.

Study	Model	Hydrogel System	Pathogen	Main Findings	References
Kaur et al. (2016)	Mouse PJI model (K-wire)	HPMC + MR-5 phage ± linezolid	MRSA	~2–3 log CFU reduction on implant and >3 log in tissue; near sterilization by day 7–10 with combination therapy; sustained in vivo phage replication (~10^6^ PFU/mL).	[59]
Wroe et al. (2020)	In vitro + murine bone defect	PEG-4MAL + phages	*P. aeruginosa*	~17-fold biofilm reduction in vitro; 4.7-fold bacterial load reduction in vivo (~10^3^ CFU/implant); sustained phage infectivity and activity.	[60]
Barros et al. (2020)	In vitro + ex vivo femur model	Alginate–nanoHA + phages	*E. faecalis* (MDR)	~99% planktonic killing; ~3-log reduction in bone; up to 99.9% inhibition of colonization; preserved osteogenic properties.	[61]
Shiue et al. (2022)	In vitro	Alginate (surface/embedded phages)	*E. coli*	~84% growth reduction (2 h); complete biofilm prevention; increased osteoblast proliferation (+28.8%) and mineralization (+43.1%).	[62]
Ferry et al. (2020)	Clinical case (DAIR)	DAC^®^ hydrogel + phages	*S. aureus*	Rapid high-titer release (~10^8^–10^9^ PFU/mL); effective infection control and successful implant retention.	[63]

**Table 4:** Summary of experimental outcomes of nanoparticle-loaded hydrogel systems in orthopedic infection models.

Study	Model	Hydrogel System	Mechanism of Action	Main Findigns	References
Wickramasinghe et al. (2020)	Mature *S. aureus* biofilms formed on clinically relevant implant materials (Ti-6Al-4V, cobalt-chromium, and tantalum).	Thermoresponsive hydrogel + AuNRs + D-AAs (PhotothermAA)	D-AAs disrupt the EPS matrix of biofilms, while AuNRs generate localized photothermal bacterial killing.	The combined AuNRs/D-AAs system achieved complete eradication of mature biofilms, whereas individual treatments resulted in residual biofilm (~15–20%).	[70]
Milbrandt et al. (2023)	Mature *S. aureus* biofilms grown on 3D-printed Ti-6Al-4V implants.		Sequential biofilm disruption by D-AAs followed by photothermal bacterial killing mediated by AuNR activation.	The combined treatment achieved complete biofilm eradication (100%) compared with approximately 25% removal using DAIR alone.	[71]
Higuera-Rueda et al. (2024)	Rabbit knee PJI model with titanium implants.		Combination of biofilm matrix disruption and photothermal antibacterial activity following laser activation.	PhotothermAA significantly reduced implant biofilm coverage (1.8% vs. 81.0% for DAIR alone, *p* < 0.0001) and decreased bacterial burden in surrounding tissues by 5.6-log CFU without tissue necrosis. These results demonstrate translational potential for implant-retention approaches in PJI.	[72]
Ding et al. (2023)	Rat implant-associated infection model.	Hyaluronic acid/gelatin hydrogel + ZIF-90-Bi-CeO_2_ nanoparticles	Bi nanoparticles provide photothermal antibacterial effects, Zn ions contribute antibacterial activity, and CeO_2_ nanoparticles reduce oxidative stress through antioxidant activity.	The hydrogel coating promoted biofilm removal, reduced inflammatory responses, and improved osseointegration. The system demonstrates the potential of multifunctional coatings combining antibacterial, immunomodulatory, and regenerative effects.	[73]
Li et al. (2026)	Titanium implant-associated infection model using a 3D-printed Ti-6Al-4V scaffold.	GelMA–tannic acid hydrogel + emodin liposomes	Liposomes provide controlled release of emodin, while emodin and tannic acid contribute antibacterial and immunomodulatory effects.	The scaffold demonstrated antibacterial activity against *S. aureus* and *E. coli* (92% and 88% inhibition, respectively), reduced biofilm formation, promoted M2 macrophage polarization, and increased bone volume fraction by 10.25%.	[74]
Du et al. (2025)	Mouse femoral fracture infection model with *Escherichia coli.*	Injectable PEG hydrogel loaded + Ångstrom-scale silver particles (AgÅPs).	Sustained local silver release provides direct antibacterial activity and reduces bacterial colonization at the infection site	Gel-AgÅPs markedly reduced viable bacterial counts recovered from the femur surface, surrounding muscle tissue, and fixation device after 7 days. After 3 weeks, treated animals remained almost sterile. The treatment preserved fracture healing in infected controls, supporting its potential for orthopedic infection management.	[75]

## Data Availability

No new data were created or analyzed in this study. Data sharing is not applicable to this article.
